# Enhancing the Stability and Catalytic Performance of Gold Subnanoclusters Mediated by Au···H–C Hydrogen Bonding and Au···π Interactions

**DOI:** 10.1021/acs.inorgchem.5c00153

**Published:** 2025-03-21

**Authors:** Alba Sorroche, Miguel Monge, José María López-de-Luzuriaga

**Affiliations:** Departamento de Química, Instituto de Investigación en Química de la Universidad de La Rioja (IQUR), 26006 Universidad de La Rioja, Complejo Científico-Tecnológico, 26006 Logroño, Spain

## Abstract

Gold subnanoclusters (AuSNCs) exhibit remarkable catalytic activity; however, their short-lived transient existence and strong tendency for self-aggregation remain disadvantageous for practical application. Considering that weak secondary interactions, such as Au···H–C or Au···π, could enhance the stability of the subnanocluster system, we have assessed their influence on the stabilization through a combination of experimental and computational analyses. We have evaluated the stabilization ability of different functional groups toward the AuSNCs system. Matrix-assisted laser desorption/ionization-time of flight (MALDI-TOF) experiments, density functional theory (DFT) calculations, and topological tools (QTAIM and NCI) provide decisive insights into the mechanism of stabilization of the short-lived AuSNCs species. Additionally, we extended the stabilization analysis to an application in catalysis. By conducting a complete NCI analysis of an optimized energy pathway, we demonstrate how an Au_3_ subnanocluster can be stabilized by a series of weak secondary interactions, including hydrogen bonds to gold (Au···H–C) as well as Au···π interactions in intermediates and transition states.

## Graphical abstract

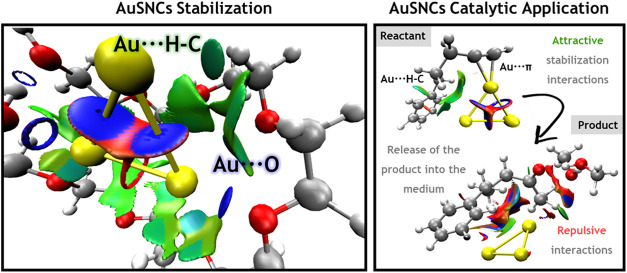

## Introduction

The last years have witnessed a significant development of gold-based molecular, nanocluster, or nanoparticle systems displaying an impressive range of applications in catalysis,^1^ medicine,^2,3^ or materials science.^4^ Some of the special characteristics and properties that gold-based systems offer arise from relativistic effects, which display a local maximum at gold.^5^ As a consequence, gold atoms display very large electronegativity, large energetic stabilization of the 6s orbital, and the destabilization of the 5d orbital. Thus, gold atoms in molecular complexes display a high ability to selectively activate π-bonds or to establish different types of dispersive-type weak interactions with other gold centers (i.e., aurophilicity) or with other atoms.^6^

The possibility of observing weak interactions between gold and hydrogen (i.e., hydrogen bonding to gold) may be expected.^7^ Indeed, recent works have dealt with the experimental and computational characterization of hydrogen bonding to gold­(I) in molecular systems,^8^ leading to the characterization of Au­(I)···H–N,^9,10^ Au­(I)···H–C,^11,12^ and Au­(I)···O–H hydrogen bonding to gold­(I).^13^ In one of these reports, the authors point that hydrogen bonding to gold­(I) can be used as a structural motif for the design of very effective catalysts.^11^ Regarding cluster-like gold species, there is no experimental spectroscopic or diffractometric evidence of hydrogen bonding to gold.^14,15^

In the same way, weak Au···π-arene interactions have also been studied experimentally and computationally for Au­(I) and Au­(III) derivatives of discrete^16^ and supramolecular nature.^17^ These interactions differ from hydrogen bonding because they are not structure-directing interactions, but contribute to the stability of the molecular systems.^17^ In contrast, the study of Au···π-arene interactions in gold clusters or nanoparticle systems is limited to computational studies.^16,18^

Ligand-free gold subnanoclusters (Au*_n_*; *n* = 3–5 or Au SNCs) constitute a very special class of subnanomaterials formed by few naked metal atoms, whose small size induces strong quantum confinement effects leading to a molecular-like behavior.^19^ The investigations in the last years have shown their high potential in photoluminescence^20,21^ and, especially, in catalysis for a broad number of interesting organic transformations. Au SNCs catalyze the formation of carbon–carbon bonds in the rearrangement of alkynylfurans leading to phenols^22^ and in the Conia-ene reaction;^23^ the formation of carbon-heteroatom bonds in the hydration of alkynes^24^ and in the bromination of aromatics or alkynes;^25^ or the formation of heteroatom-heteroatom bonds in the homocoupling of thiols.^26^ In all cases, exceptional activity and selectivity are observed.

Overall, both the possibility of establishing weak interactions between X–H or aromatic groups with Au SNCs, and the superior catalytic performance of these subnanoclusters, arise from the exposition of the metal atoms and from the availability of molecular orbitals oriented toward the surrounding environment, able to activate the organic functional groups in the catalytic transformations. However, one of the main drawbacks of Au SNCs is their short transient existence and their very high tendency for self-aggregation into nanoparticles or bulk metal. We have recently proved that in the presence of weak interactions it was possible to transiently stabilize alkyne-gold subnanocluster species.^24^ In these species, the triple bond is activated very effectively for a catalytic hydration reaction, provoking an initial stabilization of the Au SNCs. At the same time, stabilizing C–H···Au and Au···π interactions transiently prevents the aggregation of the clusters, leading to a longer lifetime for the smallest subnanoclusters and, hence, an increased catalytic activity.

We wondered whether the strength of these C–H···Au and Au···π (alkene or aromatic) interactions, alone or combined, provided by simple molecules or polymers would be enough to stabilize these Au SNCs, even in the absence of activation of the alkyne triple bond involved in a catalytic hydration transformation. To do this, we analyze herein experimentally and computationally the stabilization of gold subnanoclusters with different species that are able to provide different degrees of stabilization. For example, we chose a simple linear alkane as heptane, only able to establish C–H···Au interactions; a terminal alkene such as 5-phenyl-1-pentene leading to C–H···Au and Au···π (alkene and aromatic) interactions; an aromatic hydrocarbon such as pentylbenzene, providing C–H···Au and Au···π (aromatic) interactions; or a polymer such as poly­(ethylene glycol), bearing polyether groups and leading to C–H···Au and O···Au contacts. At the same time, we also asked ourselves whether the role played by these interactions is decisive in the formation of different intermediates and transition states in the catalytic alkyne hydration process. It is known that density functional theory (DFT) methods have been employed to gain knowledge about reaction mechanisms in catalysis; nevertheless, the topological analysis of the electron density throughout the reaction path has been less explored.^27–29^ Fewer are the examples found for organometallic catalytic systems, in which topological analysis was employed and thus, the stability of the reaction intermediates was evaluated.^30,31^ In this context, we have recently reported a DFT and topological study in which the existence of weak Au···H–X interactions constitute the driving force of an effective gold­(I)-catalyzed A^3^-coupling reaction.^32^ This type of computational analysis could manifest the presence of subtle weak interactions that could play a key role in the catalytic performance of Au SNCs.

## Results and Discussion

### Experimental Studies on the Stability of Gold(0) Subnanoclusters

Going on with our research on the transient stability of Au SNCs, we have carried out different MALDI-TOF experiments to check the stability of gold(0) subnanoclusters using different organic molecules, which can act as stabilizers of these species through weak interactions. Thus, we have evaluated this stability with 5-phenylpentyne (**1**), pentylbenzene (**2**), 5-phenylpentan-2-one (**3**), 1-hexene (**4**), and with a polyethylene glycol (**5**) polymeric matrix. The measurements were performed on aliquots from a reaction of the corresponding organic molecule in methanol at 65 °C using AuCl as source of gold(0) species after 30 min of stirring.

When stabilizing reagents are not employed, MALDI-TOF spectra reveal peaks corresponding to gold subnanoclusters up to Au_13_. Under these conditions, the Au SNCs formed from the metal precursor (AuCl) exhibit a marked tendency for self-aggregation (Figure S1). Consequently, it is expected that effective stabilization with an organic molecule would result in MALDI-TOF peaks, indicating only minimal gold self-aggregation (Au_1_–Au_5_). Conversely, inadequate stabilization would produce similar patterns to those observed in the absence of stabilizing reagent, with peaks corresponding to gold subnanoclusters up to Au_13_.

In this sense, very small gold clusters were formed using molecules (**1**) and (**5**). These stabilizing reagents revealed mass fragments that indicate the presence of Au_1_–Au_4_ species after 30 min of reaction (Figures S2 and S3). The presence of the triple bond and the phenyl group in **1** would lead to the stabilization of the gold system through Au···π interactions. Additionally, the use of a polyethylene glycol matrix also yielded promising results, likely due to the effective stabilization through Au···O and Au···H–C secondary interactions.

Furthermore, the presence of a double bond in the structure of the stabilizing molecule contributed to the successful stabilization of the gold subnanoclusters. Spectrometric analysis employing 1-hexene (**4**) as the organic stabilizer results in the detection of mass peaks corresponding to Au_1_–Au_7_ subnanoclusters (Figure S4).

In contrast with these findings, when the alkyne (**1**) was used as a structural model and its moiety was replaced with an alkane (**2**) or a ketone group (**3**), larger subnanoclusters were detected. The results revealed mass peaks corresponding to Au_1_–Au_9_ and Au_1_–Au_15_ subnanoclusters, respectively (Figures S5 and S6).

These trends reveal that an alkane (**2**) or a ketone group (**3**) is not enough to achieve effective stabilization. Consequently, the experimental results demonstrate that effective stabilization is only achieved when a Au···π interaction involving a double or a triple bond is present. Moreover, the use of a polymeric matrix provides an extraordinary method for not only stabilizing ultrasmall subnanoclusters but also effectively encapsulating them in the solid state for future applications.^33^

### Computational Analysis of the Au_3_···R Systems

#### Interaction/Dissociation Energies

After experimental evaluation of the stability of gold subnanoclusters, we also assessed their behavior through a computational analysis. The stabilization of the model system Au_3_, representing small-size Au SNCs, with various functional groups was investigated through the full optimization of the model systems at DFT-D3/M06–2X/SDD level of theory and further use of NCI, QTAIM, and NEDA topological tools. All experimental stabilizing systems were evaluated computationally through the models Au_3_···(R) (R = **1**–**5**). In addition, for the sake of comparison, we have also studied computational models Au_3_···(R) (R = **6**–**9**) as shown in Figure 1. The poly­(ethylene glycol) matrix stabilization was represented by an 8-monomer system. Moreover, we used three gold atom nanoclusters due to their stability, and to keep the calculations computationally feasible. On the other hand, and based on the experimental results, we selected the triple bond functional group for further investigations into SNCs stabilization. Thus, we constructed the model systems Au_3_-(**6**) and Au_3_-(**8**) with phenylacetylene (**6**) and 1-hexyne (**8**) as stabilizers, respectively. To assess the impact of a double or a triple bond on the AuSNCs stabilization, we replaced the triple bond in model Au_3_-(**1**) for a double bond resulting in the model Au_3_-(**7**). Finally, we examined the stabilization ability of a Au_3_ system using only alkane chain Au_3_-(**9**).

**Figure 1:**
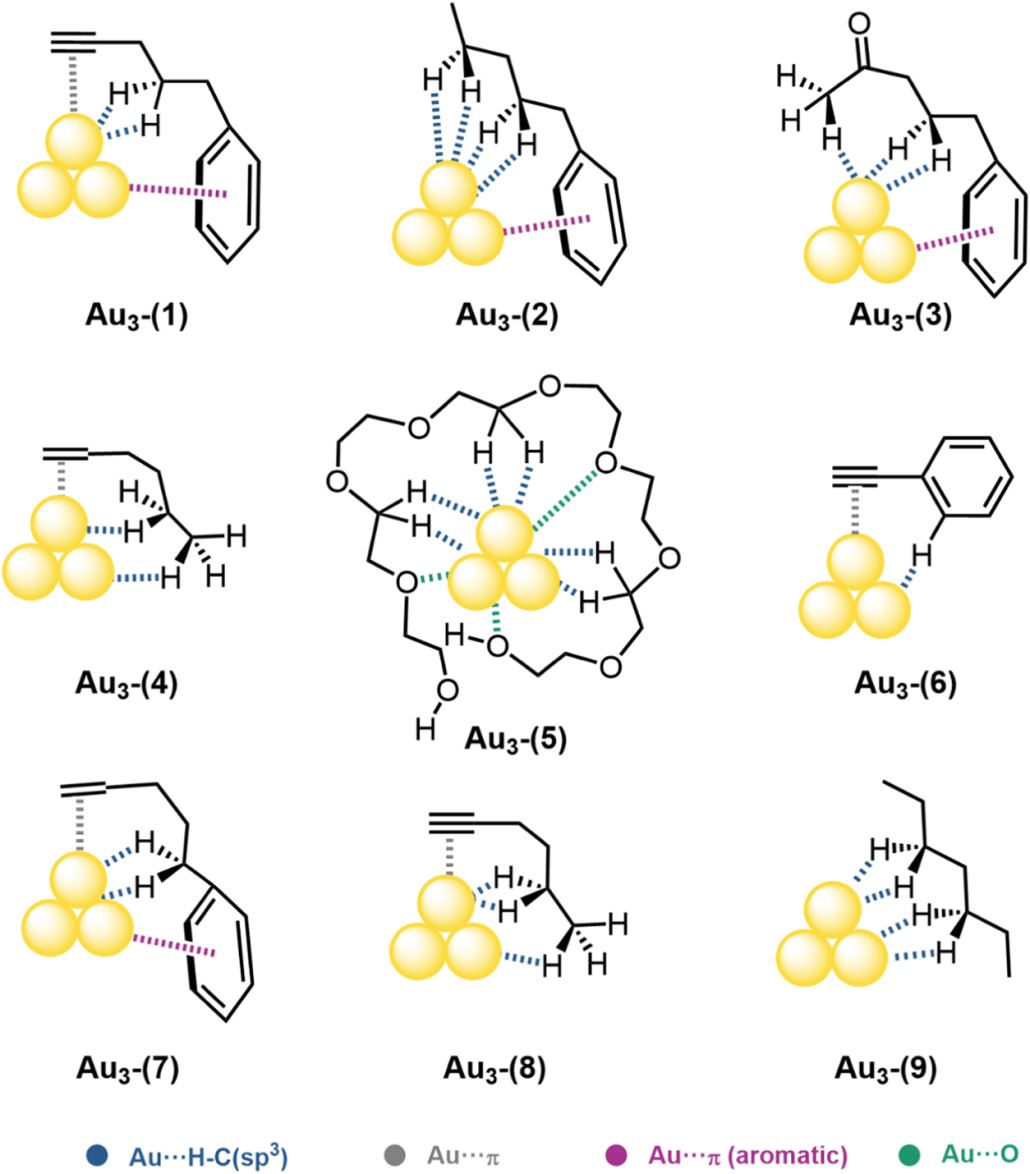
Representation of computational model systems Au_3_···R (R = **1**–**9**).

First, we evaluated the strength of the Au_3_···R interaction system by calculating the interaction and dissociation energy values (Table 1). The bonding energies were determined from optimized structures using the PBE0 functional and the def2TZVP basis sets. These interaction energies were calculated by using the counterpoise correction to the basis set superposition error (BSSE) as shown in eq 1 at the equilibrium distance between Au_3_ and the organic molecule fragments.

1 $$\Delta E_{INT}=E_{AB}^{\left(AB\right)}-E_{A}^{\left(AB\right)}-E_{B}^{\left(AB\right)}=V\left(R\right)$$

**Table 1:** Interaction and Dissociation Energies (kcal·mol^–1^) for the Optimized Model Systems

	*E*_int_	*E*_dis_
Au_3_-(**1**)	–41.5	30.5
Au_3_-out-(**1**)	–39.3	25.1
Au_3_-(**2**)	–13.4	13.3
Au_3_-(**3**)	–27.3	15.6
Au_3_-(**4**)	–43.9	31.4
Au_3_-(**5**)	–40.8	43.4
Au_3_-(**6**)	–42.6	27.0
Au_3_-(**7**)	–43.8	33.2
Au_3_-(**8**)	–41.1	28.6
Au_3_-out-(**8**)	–38.5	24.6
Au_3_-(**9**)	–13.4	11.4

The dissociation energies were calculated by using eq 2, where *E*_Au_3__ is the energy of the optimized gold nanocluster, *E*_R_ is the energy of the corresponding free ligand, and *E*_Au_3_···R_ is the energy of the total system.

$${\mathrm{Au}}_{3}\cdot \cdot \cdot R\to {\mathrm{Au}}_{3}+R$$

2 $$\Delta E_{\mathrm{dis}}=E_{{\mathrm{Au}}_{3}}+E_{R}-E_{{\mathrm{Au}}_{3}\cdot \cdot \cdot R}$$

Energy dissociation is defined as the amount of energy necessary to break a specific bond, and it is expected to be positive. Regarding this parameter, it is important to highlight a specific group of molecules that require approximately just ∼10 kcal·mol^–1^ to break the Au_3_···R interaction (Table 1). Such behavior is observed in systems stabilized by an alkyl chain Au_3_-(**9**) or an alkyl chain with a phenyl group Au_3_-(**2**), suggesting a less stable enthalpic system. A similar trend is observed in the theoretical model Au_3_-(**3**), where the ketone group does not provide any additional stabilization to the whole system resulting in the requirement of just 15.6 kcal·mol^–1^ to break the Au_3_···(**3**) interaction. On the other hand, large dissociation energies were achieved in theoretical models stabilized with molecules with double or triple bonds in their structures. These cases include the models Au_3_-(**1**), Au_3_-(**7**), and Au_3_-(**8**), whose dissociation energy values are 30.5, 33.2, and 28.6 kcal·mol^–1^, respectively. Finally, the highest dissociation energy can be found for the model Au_3_-(**5**) with a value of 43.4 kcal·mol^–1^, revealing that the Au···O interactions are highly effective in stabilizing the Au_3_ system. A comparable pattern is observed in the interaction energy values, which represent the energy released during the formation of the Au_3_···R system. This parameter is expected to be negative, indicating a thermodynamically favorable interaction. The most favorable cases are observed for the formation of the systems Au_3_-(**1**, **4**, **6**, **7**, and **8**). A common feature among the stabilizing reagents in these systems is the presence of π-density, highlighting its crucial role in the stabilizing of the AuSNCs arrangements. Finally, it is important to note that encapsulating AuSNC within a polyethylene matrix leads to a suitable environment that prevents self-aggregation and favors stabilization. In this theoretical model, Au_3_-(**5**), the combination of secondary interactions of type Au···H–C and Au···O displays favorable interaction and dissociation energies of −40.8 and 30.3 kcal·mol^–1^, respectively. Hence, polymeric matrixes, such as polyethylene glycol, are suggested as very effective materials for stabilizing unstable gold subnanoclusters.^33^

To evaluate the strength of the Au–π stabilization, Au_3_-(**1**, **8**) models were optimized avoiding the formation of weak Au···H–C secondary interactions by rotating the structure Au_3_-out-(**1**, **8**). As a result, the π stabilization alone provides favorable interaction and dissociation energy values, while the rest of weak interactions contribute to approximately ∼2 kcal·mol^–1^ to the overall stabilization of the system.

#### QTAIM and NCI Topological Analysis

A topological analysis of the electron density was also carried out on the optimized models in order to evaluate the strength and nature of the weak interactions and to prove and depict their existence.

The quantum theory of atoms-in-molecules (QTAIM) analysis over the computational model systems reveals the presence of different critical points.^34,35^ Particularly, we analyze bond critical points (BCPs), which reveal electron density regions between any pair of bonded atoms in a molecule. Several BCP descriptor parameters were analyzed, including electron density (ρ_b_), its corresponding Laplacian (∇^2^ρ_b_), as well as the kinetic (*G*_b_), potential (*V*_b_), and total electronic (*E*_b_) energy densities at the BCPs (Table S2 and Figure S14). For shared-shell interactions, the electron density is typically around 0.1 e·bohr^–3^, whereas for closed-shell interactions, such as hydrogen bonds or van der Waals interactions, the electron density ranges from 0.001 to 0.04 e·bohr^–3^. Additionally, when the value of the Laplacian is positive, the electronic density is locally reduced and relatively expanded, which corresponds to the formation of ionic, metal–metal, hydrogen, or dative bonds. In contrast, when the Laplacian value is negative, the electronic density is locally concentrated, tightly packed, and compressed, indicating the presence of covalent or polar covalent bonds.^36^ Further insights into chemical bonds can be obtained from the total electronic energy density (*E*_b_ = *G*_b_ + *V*_b_) at the BCP. Closed-shell interactions are dominated by the kinetic energy in the region of the interatomic surface, while shared interactions exhibit negative *E* values, dominated by large negative potential energy densities (*V*).^37–40^

The most significant interaction contributing to Au_3_-(**1**) stability arises from the interaction between the alkyne group and the adjacent gold atom of the cluster. QTAIM studies support this by revealing the highest electron density ρ_b_ (0.0932 e·bohr^–3^) and Laplacian ∇_ρb_^2^ (0.198 e·bohr^–5^) in this region (Table S2). In addition, the only negative value for total electronic energy *E*_b_ (−0.0291 e·bohr^–3^) has been found in this region. Although the ρ_b_ value is close to the ones expected for shared electron interactions, the sign of the Laplacian suggests the presence of a closed-shell interaction. Within all of these interactions, the sign of the electronic energy indicates the existence of a dative bond, specifically indicating a Au–π dative bond. This behavior is consistently observed across other model systems containing an alkyne group within the stabilizing reagent structure Au_3_-(**6**, **8**). It is worth noting that Au_3_-(**6**) model only shows one BCP indicating a Au–π bonding, with electron density ρ_b_, Laplacian ∇_ρb_^2^, and electronic energy values of 0.0991 e·bohr^–3^, 0.2218 e·bohr^–5^ and −0.0323 e·bohr^–3^ (Table S2), respectively. Despite this, the interaction (−42.6 kcal·mol^–1^) and dissociation energies (27.0 kcal·mol^–1^) are enough to demonstrate a strong and stable interaction.

Alternatively, we can find other types of interactions between a Au_3_ subnanocluster and the stabilizing molecule of the system in the Au_3_-(**1**) model. The analysis reveals weak interactions of type Au···H–C­(sp^3^) with electron density values in the range of ρ_b_ = 0.0088–0.00739 e·bohr^–3^, ∇_ρb_^2^ = 0.026–0.018 e·bohr^–5^, and *E*_b_ = −0.0005–(−0.0010) e·bohr^–3^ (Table S2). They are located in the C­(sp^3^)–H···Au gradient density path that connects gold atoms with the hydrogen atom of the alkyne chain. Those values for the QTAIM parameters suggest weak closed-shell interactions, consistent with hydrogen bond characteristics.

To evaluate the stability of Au_3_···alkyne systems, we built a model Au_3_-(**2**) by replacing the alkyne group of Au_3_-(**1**) with an alkyl chain. The results indicate that the Au···π interaction involving the alkyne group plays a crucial role in the stability of the whole model system. Consistently, in the case of model Au_3_-(**2**), where the Au–π interaction is absent, other type of weak interactions emerge, resulting in an interaction energy of −13.4 kcal·mol^–1^. QTAIM analysis reveals the presence of a BCP along the density path between Au and H–C­(sp^3^), showing ρ_b_ values ranging from 0.0076 to 0.0308 e·bohr^–3^ and ∇_ρb_^2^ values ranging from 0.018 to 0.093 e·bohr^–5^ (Table S2). The strongest interaction is found in a Au···H–C­(sp^3^) interaction with a Au···H distance of 2.309 Å. However, all of these weak interactions of Au···H–C­(sp^3^) are not enough to produce an interaction energy (−13.4 kcal·mol^–1^) stronger than that observed for the Au_3_-(**1**) (−41.5 kcal·mol^–1^) model system. Changing to the ketone stabilizing reagent (**3**), few BCPs were found, with the strongest occurring between the gold atom and the π density of the aromatic group, characterized by values of 0.0525 e·bohr^–3^ (ρ_b_) and 0.132 e·bohr^–5^ (∇_ρb_^2^).

Furthermore, QTAIM analysis over computational models Au_3_-(**4**) and Au_3_-(**7**) reveals a similar electronic density of 0.0917 and 0.0907 e·bohr^–3^, respectively, at the BCP located between gold and the π density of the double bond. In addition to the negative signs of the electronic energy values, this result indicates that the double bond also plays a significant role in stabilizing the gold subnanocluster system through a Au–π dative interaction.

In the case of the Au_3_-(**5**) model, the stabilization of ultrasmall subnanoclusters is significantly enhanced by numerous Au···H–C­(sp^3^) interactions, along with the combined contribution of Au···O interactions, despite the absence of Au–π interactions.

In addition, NCI topological analysis is a highly effective tool for graphically illustrating the presence of noncovalent interactions in the real space.^41–43^

A qualitative classification of the noncovalent interactions can be derived by analyzing the sign of Laplacian of the electron density. This parameter can be represented as a sum of three eigenvalues of the electron density Hessian (second derivative) matrix: ∇^2^ρ = λ_1_ + λ_2_ + λ_3_ (λ_1_ ≤ λ_2_ ≤ λ_3_). A thorough analysis of the sign of the second λ_2_ eigenvalue is essential to distinguish different types of noncovalent interactions. When the sign of the second Hessian eigenvalue is positive, steric and repulsive interactions are identified and red isosurfaces appear. If the sign of λ_2_ is close to zero, van der Waals weak interactions are assigned, and green isosurfaces are observed. Finally, when the sign of the second Hessian eigenvalue is negative, attractive contacts are assigned and blue isosurfaces can be observed.

NCI analysis can be visually represented in two modes: 2D graphical plots and 3D isosurfaces. The 2D plot displays the reduced density gradient against the product of the electron density and the sign of the second Hessian eigenvalue (sign­(λ_2_)­ρ). The 3D isosurfaces provide a special representation of noncovalent interactions in the real space, displaying the distribution of these interactions as broad regions. We conducted this analysis on model systems Au_3_-(**1**–**9**) as shown in Figure 2. The resulting topological surfaces graphically depict the presence and nature of weak interactions within the described models. In Au_3_-(**1**) model, weak van der Waals interactions depicted by green surfaces reveal Au···H–C­(sp^3^) interactions between the gold subnanocluster and the aliphatic chain or Au···H–C­(sp^2^) with the aromatic ring. Figure 2 clearly depicts the presence of weak van der Waals interactions through green isosurfaces in the Au_3_-(**1**) model and the absence of these interactions in the Au_3_-out-(**1**) model. The most notable result arising from these topological calculations is observed in the Au_3_-(**5**) model system. Green surfaces indicate the presence of Au···H–C­(sp^3^) interactions, while blue surfaces, representing stronger attractive interactions, appear between the gold subnanocluster and the oxygen atoms of the ether groups in the 8-monomer polymeric model, showing that Au···O contacts efficiently contribute to the stabilization of the Au_3_ SNCs (Figure 3).

**Figure 2:**
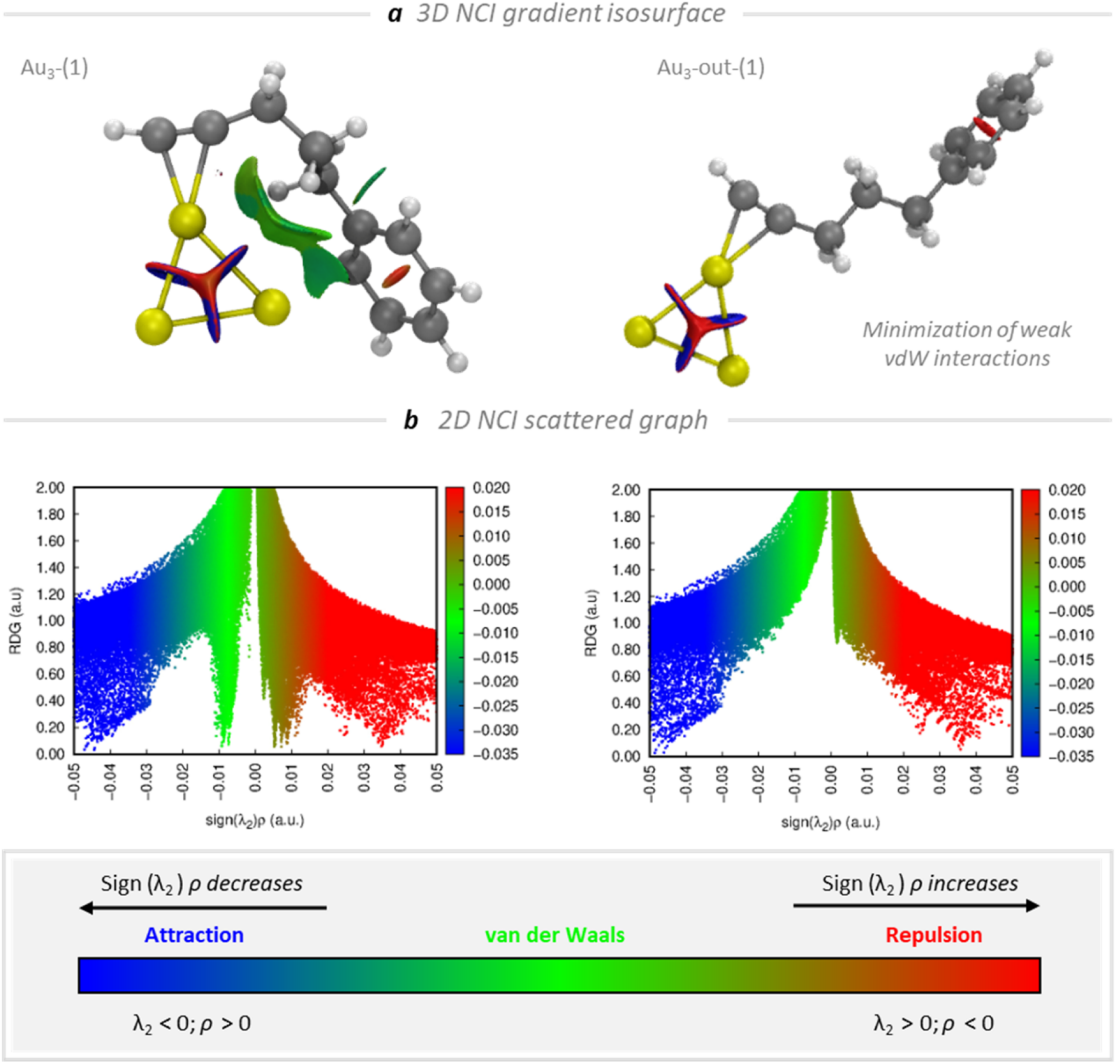
NCI analysis of the optimized Au_3_-(**1**) and Au_3_-out-(**1**) theoretical models. (a) 3D-NCI gradient isosurface plots (isovalue 0.4 au) and (b) 2D-NCI scattered graphs.

**Figure 3:**
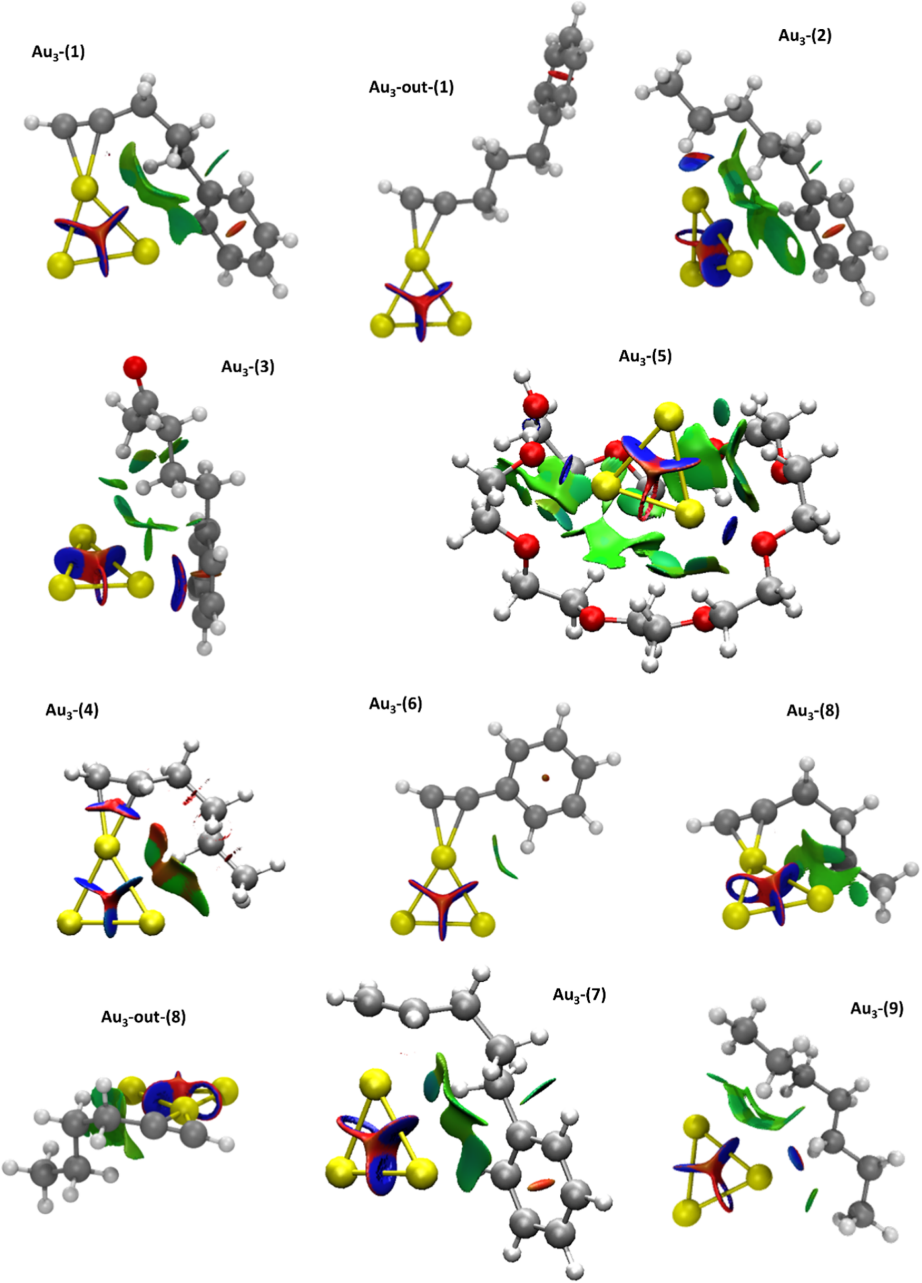
NCI analysis of the optimized Au_3_-(**1**–**9**) theoretical models. The isovalue is 0.4 au and the color scale of −3 < ρ > 3.

#### NEDA Analysis

To better understand the stabilization of Au subnanoclusters and the type of interaction energies involved in the transient stability of the Au_3_···R systems, we performed natural energy decomposition analysis (NEDA) to the electron density of the computational models described in this study. The results are listed in Table S3. This analysis provides a segregation of the system interaction energies (*E*_INT_) between the gold subnanocluster and the organic molecule including electrical (*E*_EL_) interaction, charge transfer (*E*_CT_), and core repulsion (*E*_CORE_).

For model Au_3_-(**1**), charge transfer *E*_CT_ (−110.6 kcal·mol^–1^) is the most dominant contribution to the attractive interactions followed by the electrical energy *E*_EL_ (−87.6 kcal·mol^–1^). These attractive interactions supplied the *E*_CORE_ repulsion (164.3 kcal·mol^–1^) between the gold subnanocluster and the organic molecule are enough to stabilize the whole system (−33.9 kcal·mol^–1^). The stabilization of this system is primary achieved through charge transfer from the π density of the alkyne to the gold atom of the subnanocluster. However, when the Au–π bonding interaction does not exist, the sum of the attractive energies is not enough to overcome the repulsion interactions of the core. This is clearly evident in models Au_3_-(**2**,**9**), which display *E*_int_ values of −7.5 and – 4.7 kcal·mol^–1^, respectively, and where the gold subnanoclusters are stabilized only by aliphatic chains.

On the other hand, when weak interactions were minimized in Au_3_-out-(**1**) computational model and was compared to Au_3_-(**1**), the *E*_CORE_ repulsion is reduced (151.8 kcal·mol^–1^), along with the charge transfer *E*_CT_ (−105.5 kcal·mol^–1^). This loss of weak interactions is also reflected in the decrease in the electrostatic *E*_ES_ component (−72.0 kcal·mol^–1^). This trend is also observed in the hexyne models Au-(**8**) and Au-out-(**8**).

The results also show that the use of different alkynes is not highly significant; however, it is worth noting that the Au_3_-(**6**) model exhibits a higher charge transfer energy compared to models with 5-phenylpentyne (**1**) or hexyne (**8**). It appears that this more rigid system favors a larger *E*_CT_ component.

NEDA analysis reveals other remarkable *E*_INT_ values of −35.4 and −38.2 kcal·mol^–1^ found for Au_3_-(**7**) and Au_3_-(**4**) models, respectively, with the primary interaction occurring between the gold subnanocluster and the alkene functional group. The interaction energy is also high (−25.4 kcal·mol^–1^) for the model stabilized with polymer Au_3_-(**5**).

In view of *E*_ES_, which considers the interaction between two fragments of the system before the formation of the molecule, it is higher when Au–π or Au···O interactions are shown.

### Free Energy Catalytic Pathway Analysis

Considering the experimental and computational results described above, we have chosen alkynes as the most suitable substrates for the stabilization of the gold subnanocluster and, concomitantly, for their use as reagents for hydration catalytic reactions. Based on previous studies, we assessed the catalytic activity of AuSNCs generated in situ of the hydration reaction of several alkynes (Figure 4). By examining both the catalytic efficiency and the formation of different species of AuSNCs, we evaluated the AuSNCs stabilization efficiency of the selected alkynes included in this the study. Thus, very different results were observed when the alkyl chain or the functional group was modified.

**Figure 4:**
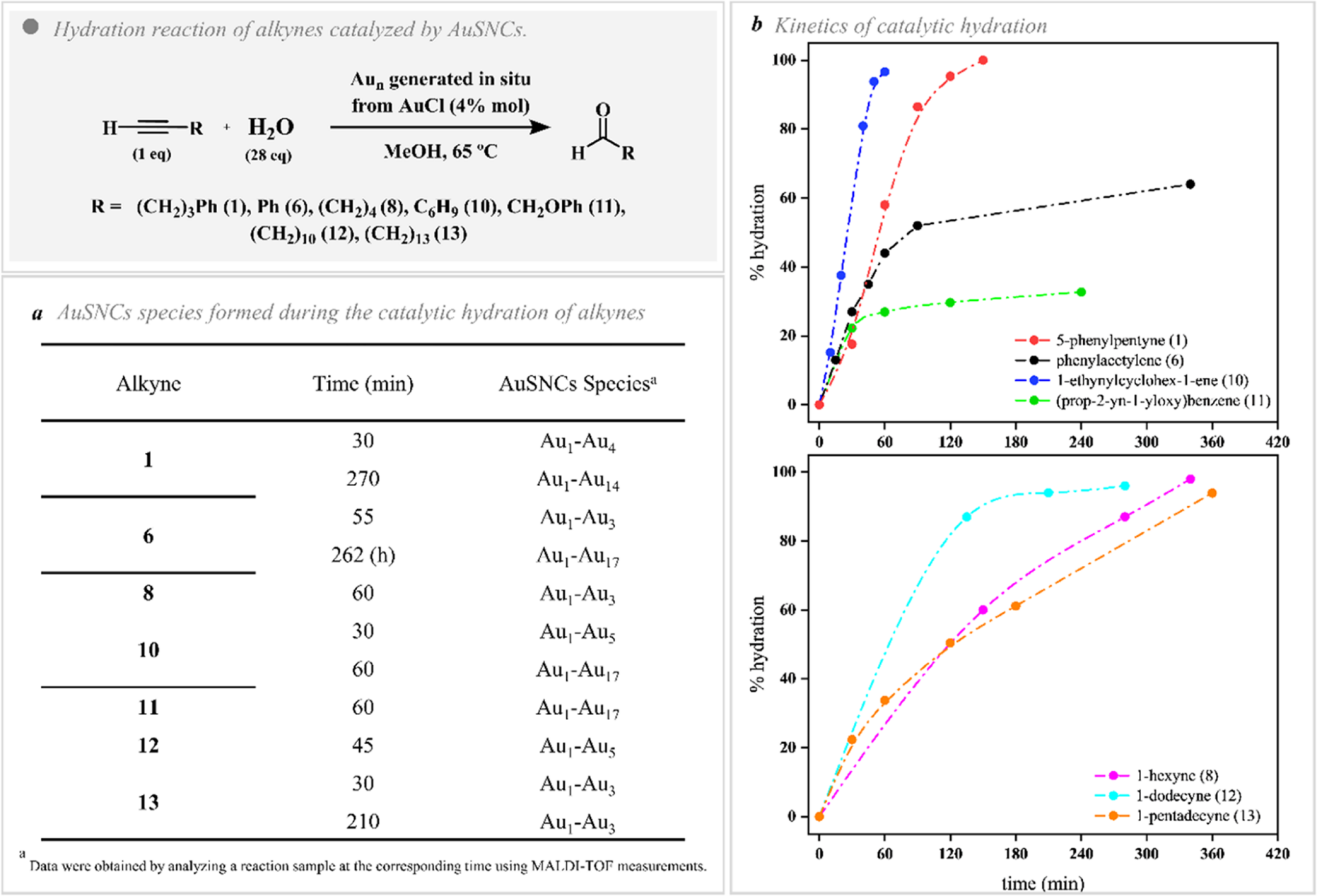
Experimental data of AuSNC catalyzed hydration reaction of alkynes. (a) MALDI-TOF Au*_n_* peaks observed during the catalytic performance. (b) Kinetics of the catalytic reaction for: Alkynes with phenyl groups 5-phenylpentyne (**1**), phenylacetylene (**6**), 1-ethynylcyclohex-1-ene (**10**), and (prop-2-yn-yloxy)­benzene (**11**) (top). Alkynes with aliphatic chains 1-hexyne (**8**), 1-dodecyne (**12**), and 1-pentadecyne (**13**) (bottom).

The catalytic experiments were performed by placing 9.4 mg of AuCl (0.04 mmol) into a round-bottom flask with 5 mL of methanol as solvent. Then, 500 μL of water and the corresponding alkyne (1 mmol) were added, and the mixture was magnetically stirred at 65 °C for the required time in each case. The catalytic reaction was monitored via GC-MS chromatography. The highest catalytic efficiency is observed for the alkynes (**1**) and (**10**), achieving hydration conversions of >95% in 2 and 1 h, respectively (Figure 4b, top). Interestingly, MALDI-TOF measurements revealed that during the initial stages of the reaction, small AuSNCs were detected. However, when the reaction is almost complete, those species developed into larger AuSNCs, specifically Au_14_ and Au_17_, respectively (Figure 4a). This result probably reveals the fact that these alkynes are effective enough to stabilize small subnanoclusters, but when the hydration is almost completed and the ketone product is released into the medium, the product (ketone) appears to be less effective as a stabilizer compared to the alkyne reagent. Consequently, the catalytic species would undergo self-aggregation, leading to the formation of larger AuSNCs. This agrees with the stabilization analysis described in this study (vide supra), which shows that the loss of the alkyne π-system in a ketone structure Au_3_-(**3**) does not provide a strong interaction that effectively stabilizes the Au_3_ system as, for instance, in Au_3_-(**1**).

On the other hand, alkynes with different alkyl chains were evaluated (**8**, **12**, and **13**) (Figure 4b, bottom). In this sense, by taking 1-hexyne (**8**) and 1-dodecyne (**12**) alkynes, a larger chain achieves better hydration conversions. However, when the 15-carbon alkyne (**13**) is used, worse results are observed compared to the six-carbon alkyne (**8**). In this sense, it is important to note that stabilizing the AuSNCs is just as crucial as facilitating the catalytic reaction by leading the reactants to be close enough to produce the reaction. Thus, the lower catalytic efficiency observed for the 15-carbon alkyne (**13**) could be attributed to the too large stabilization of the Au SNCs, preventing their catalytic cycling with further alkyne molecules. Accordingly, small AuSNCs were observed in mass spectrometry results even in advanced steps of the catalytic hydration demonstrating its stabilizing effectiveness, i.e., Au_3_ species were detected even after 210 min of the hydration of compound **13**.

Finally, the worst hydration efficiency is observed for phenylacetylene (**6**) and (prop-2-yn-1-yloxy)­benzene (**11**) compounds. MALDI-TOF measurements on the reaction with **11** reveal self-aggregation since the beginning of the reaction (60 min) showing AuSNCs up to Au_17_ species. The presence of these larger species does not provide a triple bond activation, leading to poor catalytic results. On the other hand, phenylacetylene (**6**), as we have pointed out in this study, does not exhibit weak interactions that could stabilize the Au_3_ species. Hence, the catalytic efficiency revealed poor results. The mass spectrometry analysis only reveals some self-aggregation (Au_1_–Au_17_) after 262 h of reaction. This could be interpreted in terms of a very fast aggregation of Au SNCs at the beginning of the reaction, leading to Au NPs, from which Au SNCs species would be slowly released under catalytic reaction conditions and long reaction times.

To gain more insights into the catalytic activity of this gold(0) species and the role played by noncovalent interactions throughout the entire catalytic process, we have carried out a computational study of an alkyne hydration reaction using DFT methods and Au_3_-(**1**) as a model system.^24^ A three gold atom subnanocluster has been established as the most convenient subnanocluster for this type of calculations.^19,44^

The catalytic cycle was proposed for other alkyne molecules in previous studies.^24^ We calculated the complete minimum-energy reaction pathway for the proposed mechanism employing two implicit methanol molecules supporting the stabilization of the whole system. Moreover, bulk solvent methanol effects were considered by using the SMD solvation model.^45^

In all steps of the catalytic profile, NCI calculations were performed to assess the influence and type of noncovalent interactions that may emerge or disappear during the process, stabilizing intermediates and transition states, and contributing to the formation of the final ketone compound (Figures 5 and S16).

**Figure 5:**
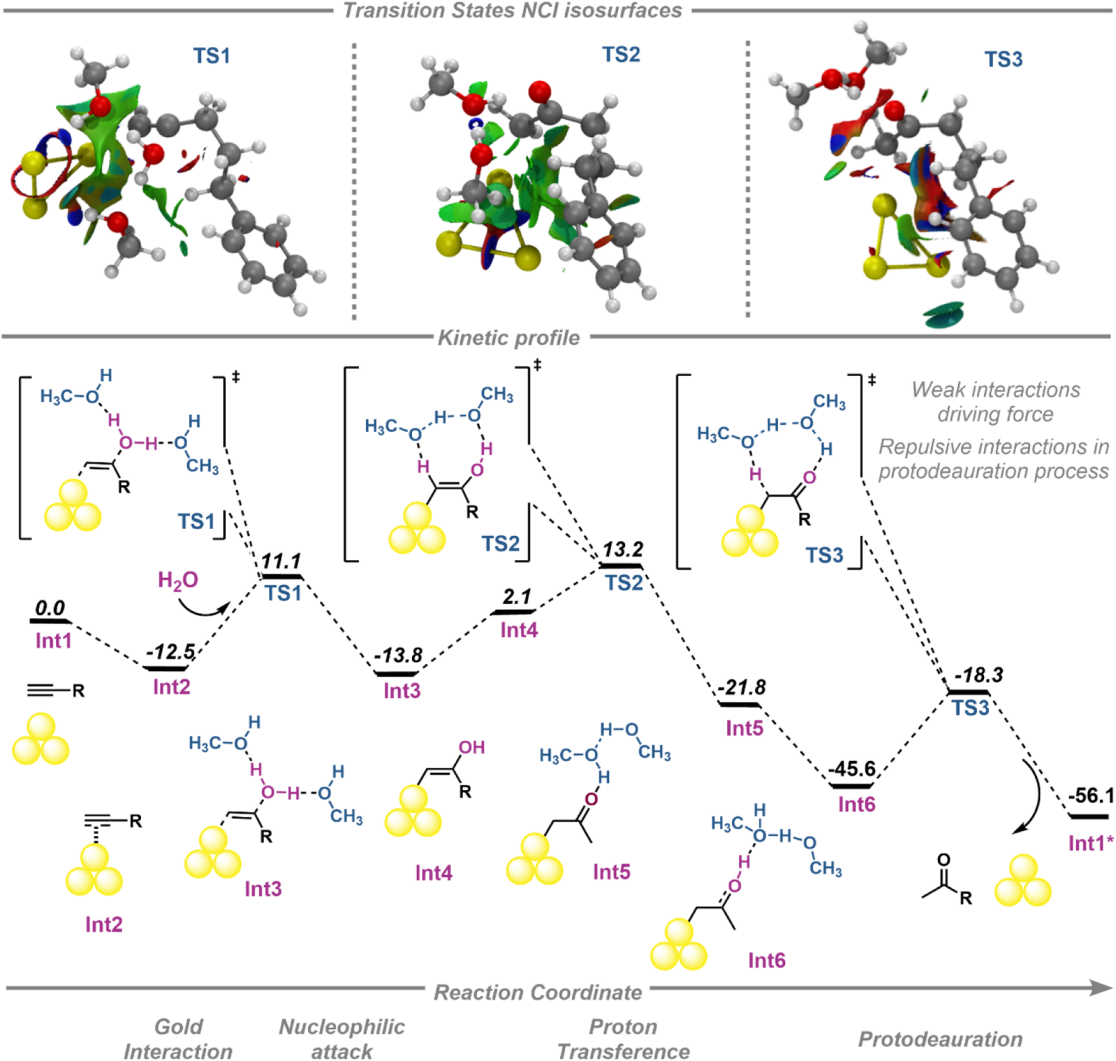
Complete minimum-energy reaction pathway calculated for the proposed mechanism of alkyne hydration catalyzed by gold subnanoclusters at the M062X level. Gibbs free energies are given in kcal·mol^–1^. NCI isosurface of the optimized transition states showing Au···H–C interactions (isovalue = 0.6). Color code: Au, yellow, yellow, O, red, C, gray, H, white.

The first stage of the reaction path, **int2** represents the interaction of the alkyne with the subnanocluster. Hereafter, we find the first transition state (**TS1**) of the reaction path which requires 23.6 kcal·mol^–1^ to its formation. In this transition state, weak interactions are observed between the π density of the alkyne and the Au_3_ subnanocluster, assisted by two methanol molecules. All van der Waals weak interactions contribute to the final stability of the whole system. Following the catalytic profile, a tautomeric equilibrium was established between the keto–enol forms. In one of the intermediates (**int4**), the NCI calculations show a broad van der Waals weak interaction surface region between the gold subnanocluster and the aromatic density of the substrate ring, resulting in the transient stabilization of the less stable gold naked catalysts species (Figure S16). In the next steps of the catalytic profile, hydrogen migration from the enol tautomeric form produces the final ketone. These steps involve the transition states **TS2** and **TS3** with energy barriers of 11.1 and 27.3 kcal·mol^–1^, respectively. Moreover, during the hydrogen migration, weak interactions established between the solvent and the Au_3_···alkyne system highlight the importance of the solvent in the stabilization of the overall system.

Finally, during the protodeauration step, the NCI isosurface provides evidence of ketone formation and its release in the medium. This is represented by the absence of the Au–π bond and the presence of the repulsive interactions (red color surfaces) between the gold catalyst subnanocluster and the final product of the hydration catalysis (**TS3**, Figure 5). Once the subnanocluster is released into the medium, it may undergo self-aggregation, as this process is highly thermodynamically favored. The NCI results on this transition state of the catalytic cycle are consistent with both experimental and computational results obtained for the Au_3_-(**3**) system. The observed inefficient stabilization ability of 5-phenylpentan-2-one (**3**) in the Au_3_-(**3**) system is characterized by the formation of larger subnanoclusters up to Au_15_ due to aggregation, along with unfavorable computed interaction/dissociation energies (e.g., dissociation energy of 15.6 kcal·mol^–1^), which aligns with the TS3 and the release of the ketone into the medium. Experimental results obtained from the catalytic hydration reactions agree with these computational findings, as small AuSNCs were observed in the first stages of the reaction, followed by a self-aggregation process (Au_1_–Au_17_) when the ketone product is not effective enough to stabilize the AuSNCs species.

If we compare the catalytic profile of alkynes with different structures, considering mainly the length of the alkyl chain, it is clearly observed that the increasing number of carbon atoms in the chain causes a less energetically demanding catalytic profile (Figure S17). Otherwise, if we attend only to the topological analysis and the interaction energy value calculated for the Au_3_-(**6**), which corresponds to the **int2** geometry, we can find it to be quite similar to that of the Au_3_-(**1**) model (−42.6 and −41.5 kcal·mol^–1^, respectively, Table 1). This suggests that the dissociation energy values alone do not fully account for the catalytic performance of the gold subnanocluster catalysts studied in this work.

## Conclusions

In summary, we have demonstrated the crucial role played by hydrogen to gold interactions (Au···H–C) and Au···O weak interactions along with Au–π bonding interactions, in stabilizing the short-lived gold subnanocluster species. Experimental MALDI-TOF measurements revealed how different organic molecules provide varying degrees of subnanocluster stabilization, as indicated by the presence of different Au*_n_* *m*/*z* peaks.

The computational analysis offers essential insights into how weak interactions can provide the necessary environment to increase the lifetime of the short-lived AuSNCs, offering a clue to finally achieve effective stabilization on AuSNCs.

Experimental work demonstrates that the better catalytic efficiency correlated with better AuSNCs stabilization, as indicated by the Au*_n_* peaks observed in MALDI-TOF analysis. Computational calculations on minimum-energy pathways support experimental evidence, showing that Au_3_ stabilization occurs through weak interactions throughout all of the energetic profile, including the transition states. This continues until the formation of the final ketone, where the NCI analysis reveals repulsive interactions between the molecule and the Au_3_ SNCs, leading to the release of the product.

Our work offers valuable insights into how to stabilize gold subnanocluster temporary species for their use in different catalytic reactions. Additionally, Au···π interactions and weak Au···H–C interactions constitute a key factor in the design of new catalytic systems. These interactions are crucial not only for stabilizing the reactive and less stable gold subnanoclusters but also for the design of novel gold-based species able to catalyze other organic reactions.^46^

## Experimental Section

### General Considerations

The subnanocluster precursor AuCl was obtained from Strem Chemicals, Inc. and used as received. The reagents used as stabilizers and for the catalytic experiments were purchased from Fluorochem and Alfa Aesar. The poly­(ethylene glycol) matrix was obtained from Sigma-Aldrich with a M.W. of 3400.

### Instrumentation

MALDI-TOF spectra were recorded on a Microflex MALDI-TOF Bruker spectrometer. The samples were prepared by adding 1 μL of the reaction mixture to a hole of an MSP ground Steel BC sample holder (Bruker). The samples were dried before the measurements.

The quantitative monitoring of the catalytic reaction was performed by gas chromatography using an Agilent 8890 GC System, equipped with a J&W HP-5 ms Ultra Inert GC Column (30 m × 0.25 mm × 0.25 μm) and MS detector (electron impact with a single quadrupole filter). A split injection system with a split ratio of 100:1 was used with helium as carrier gas at a head pressure of 10.4 psi. The temperature programming was 40 °C/min, 180 °C (3 min) and 60 °C/min (5 min), 300 °C (7 min). The conversion of the alkyne reactive and the ketone product yield were analyzed by integrating the chromatographic peaks of 5-phenylpentyne (retention time 3.165 min) and 5-phenylpentan-2-one (retention time 3.750 min). The same procedure was taken for the alkynes **6**, **8**, **10**, **11**, **12**, and **13**.

### Catalytic Experiments

In a typical catalytic hydration experiment, 9.4 mg of AuCl (0.04 mmol) was placed in a round-bottom flask. Methanol (5 mL), 500 μL of water, and the corresponding organic stabilizing molecule were added. The mixture was magnetically stirred at 65 °C for the time required in each case.

### Computational Details

The optimizations of all model systems included in this study were computed at the DFT level of theory using the M06–2X hybrid functional^47^ within the Gaussian 16 package program.^48^ In addition, solvent effects (CH_3_OH) were considered. We selected the M06–2X hybrid functional for the whole study since it has been proved before by our group in a similar analysis. The heteroatoms were treated by SDD pseudopotentials,^49^ including only the valence electrons for each atom. For these atoms, double-ζ basis sets were used, augmented with d-type polarization functions.^50^ For H atoms, a double-ζ basis set was used, together with a p-type polarization function.^51^ The 19-valence electron SDD pseudopotential^52^ was employed for Au atoms, together with two f-type polarization functions.^53^ In this way, we have optimized the models of the species Au_3_-(**1–9**) (5-phenylpentyne (**1**), pentylbenzene (**2**), 5-phenylpentan-2-one (**3**), 1-hexene (**4**) and with a polyethylene glycol (**5**), phenylacetylene (**6**), pent-4-en-1-ylbenzene (**7**), 1-hexyne (**8**) and heptane (**9**)) and Au_3_-out-(**1**, **8**). We used three gold atom nanoclusters due to their stability and to keep the calculations computationally approachable.

The bonding energies were carried out from the optimized structures employing the PBE0 ^54–57^ functional and the def2TZVP basis set.^58–61^ These energies were calculated by using the counterpoise correction to the basis set superposition error (BSSE)^62,63^ (eq 3) at the equilibrium distance between [Au_3_] and the organic molecule fragments.

1a $$\Delta E_{\mathrm{INT}}=E_{\mathrm{AB}}^{\left(\mathrm{AB}\right)}-E_{A}^{\left(\mathrm{AB}\right)}-E_{B}^{\left(\mathrm{AB}\right)}=V\left(R\right)$$

The dissociation energies were calculated by using eq 4, where *E*_Au_3__ is the energy of the optimized gold nanocluster, *E*_R_ is the energy of the correspondent free ligand, and *E*_Au_3_···R_ is the energy of the total system.

$${\mathrm{Au}}_{3}\cdot \cdot \cdot R\to {\mathrm{Au}}_{3}+R$$

2a $$\Delta E_{\mathrm{dis}}=E_{{\mathrm{Au}}_{3}}+E_{R}-E_{{\mathrm{Au}}_{3}\cdot \cdot \cdot R}$$

In addition, we have also performed a topological analysis on each theoretical model, with the main objective of analyzing the chemical nature of bonding from a qualitative point of view. The electron density ρ­(*r*) of the fully optimized structures using QTAIM analysis and the NCI analysis was computed by Multiwfn software^64^ on wfn files which were built up with TURBOMOLE version v 6.4 ^65^ using PBE0-D3/def2TZVP level of theory. We have also performed the natural energy decomposition analysis (NEDA) on the M06–2X optimized structures with NBO7.^66^ This bonding analysis was carried out at the PBE0/def2TZVP level of theory. QTAIM and NCI representations were performed with VMD visualization package program.^67^

The NEDA analysis decomposes the interaction energy between selected fragments into the electrical interaction (*E*_EL_), charge transfer (*E*_CT_), and core repulsions (*E*_CORE_) components (eq 3).

3 $$E_{\mathrm{INT}}=E_{\mathrm{EL}}+E_{\mathrm{CT}}+E_{\mathrm{CORE}}$$

The electrical interaction (*E*_EL_) is a stabilizing component represented by the sum of the electrostatic component (*E*_ES_) and the polarization effects which includes the polarization component (*E*_POL_) and the self-polarization energy of the components (*E*_SE_) (eq 4)­

4 $$E_{\mathrm{EL}}=E_{\mathrm{ES}}+E_{\mathrm{POL}}+E_{\mathrm{SE}}$$

The core repulsion component is represented by the sum of the Pauli repulsion (*E*_DEF_), electron exchange, and correlation effects (*E*_XC_) minus the self-polarization energy of the components (*E*_SE_) (eq 5)­

5 $$E_{\mathrm{CORE}}=E_{\mathrm{DEF}}+E_{\mathrm{XC}}-E_{\mathrm{SE}}$$

On the minimum-energy catalytic profile, all geometry optimizations and transition structure searches were carried out with the Gaussian 16 package program. Solvent methanol effects were considered by using the SMD solvation model.^45^ In these calculations, two molecules of methanol were included in order to assist the hydration of the alkyne. In addition, the NCI surfaces were carried out throughout the catalytic profile.

## Supplementary Material



## Notes

Additional experimental procedures, MALDI-TOF spectra, and computational methods (PDF)

## Abbreviations

SNC: subnanocluster
