# Supplementary material for: Enhancing the Stability and Catalytic Performance of Gold Subnanoclusters Mediated by Au···H–C Hydrogen Bonding and Au···π Interactions
Source: Inorg Chem. 2025 Mar 21;64(12):6301–12. doi: 10.1021/acs.inorgchem.5c00153 (PMC12179818; doi:10.1021/acs.inorgchem.5c00153)
Supplement: Supplementary file 1 [file ic5c00153_si_001.pdf]

Supporting Information

Enhancing the Stability and Catalytic Performance of Gold Subnanoclusters Mediated by Au···H-C Hydrogen Bonding and Au···π Interactions

Alba Sorroche, Miguel Monge and José María López-de-Luzuriaga**

Departamento de Química, Instituto de Investigación en Química de la Universidad de La Rioja (IQUR), Universidad de La Rioja, Complejo Científico-Tecnológico, 26006, Logroño, Spain.

E: mail: miguel.monge@unirioja.es; josemaria.lopez@unirioja.es

Table of contents

1. Experimental Spectra 2

2 Computational Analysis 12

2.1 Results and discussion 13

2.2 Cartesian coordinates of the lowest energy calculated structures 21

1. Experimental Spectra

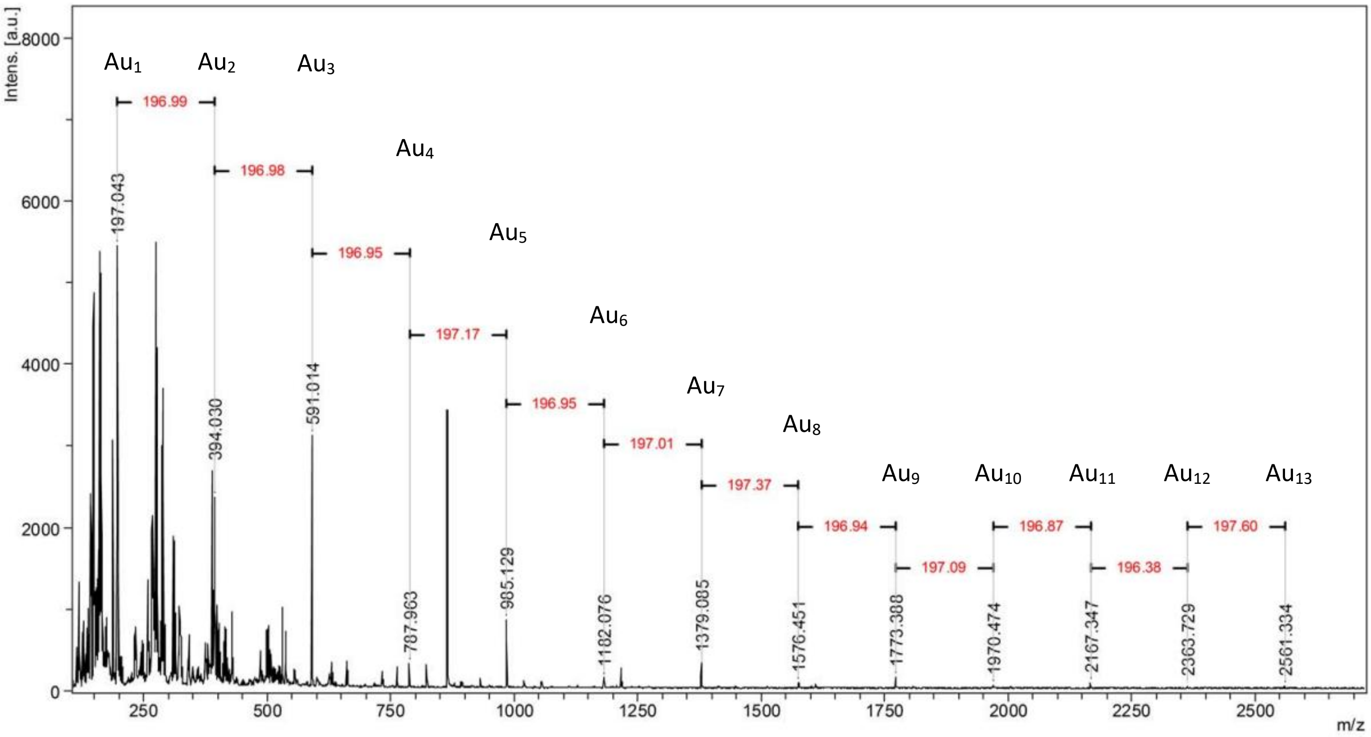

Figure S1. MALDI-TOF spectra showing the formation of larger size gold clusters at 30 minutes of reaction conditions. No stabilizer agent was used in this case.

a

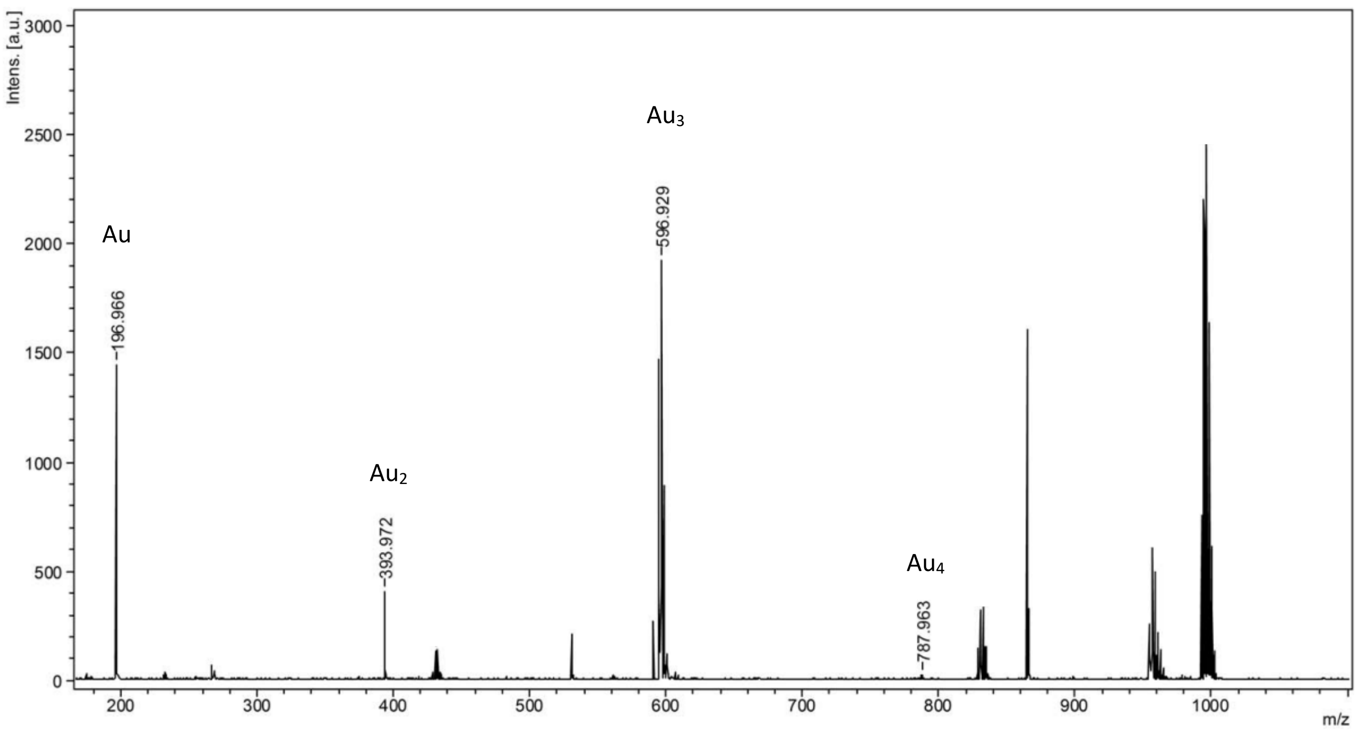

b

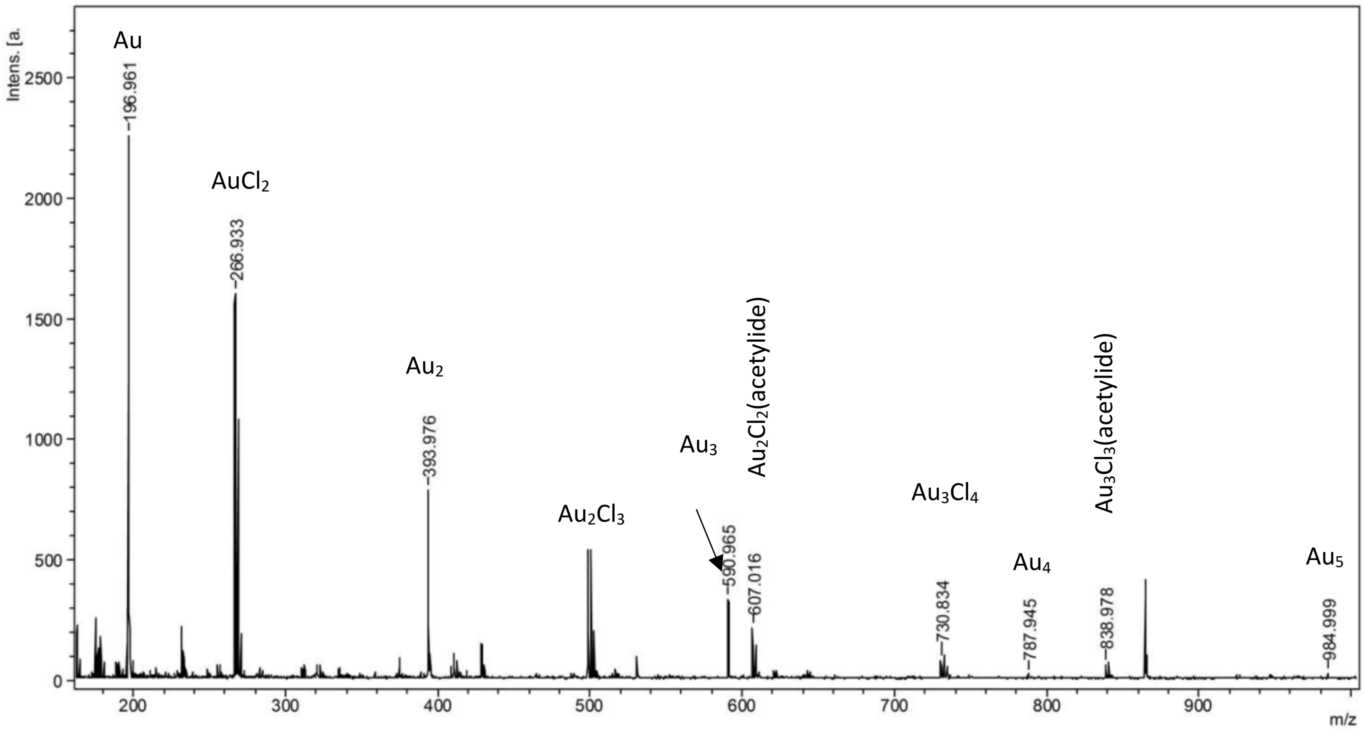

Figure S2. MALDI-TOF spectra showing the formation of small size gold clusters at **a,** 15 minutes and **b,** 150 minutes at the reaction conditions, using 5-phenylpentyne (**1**) as subnanocluster stabilizer.

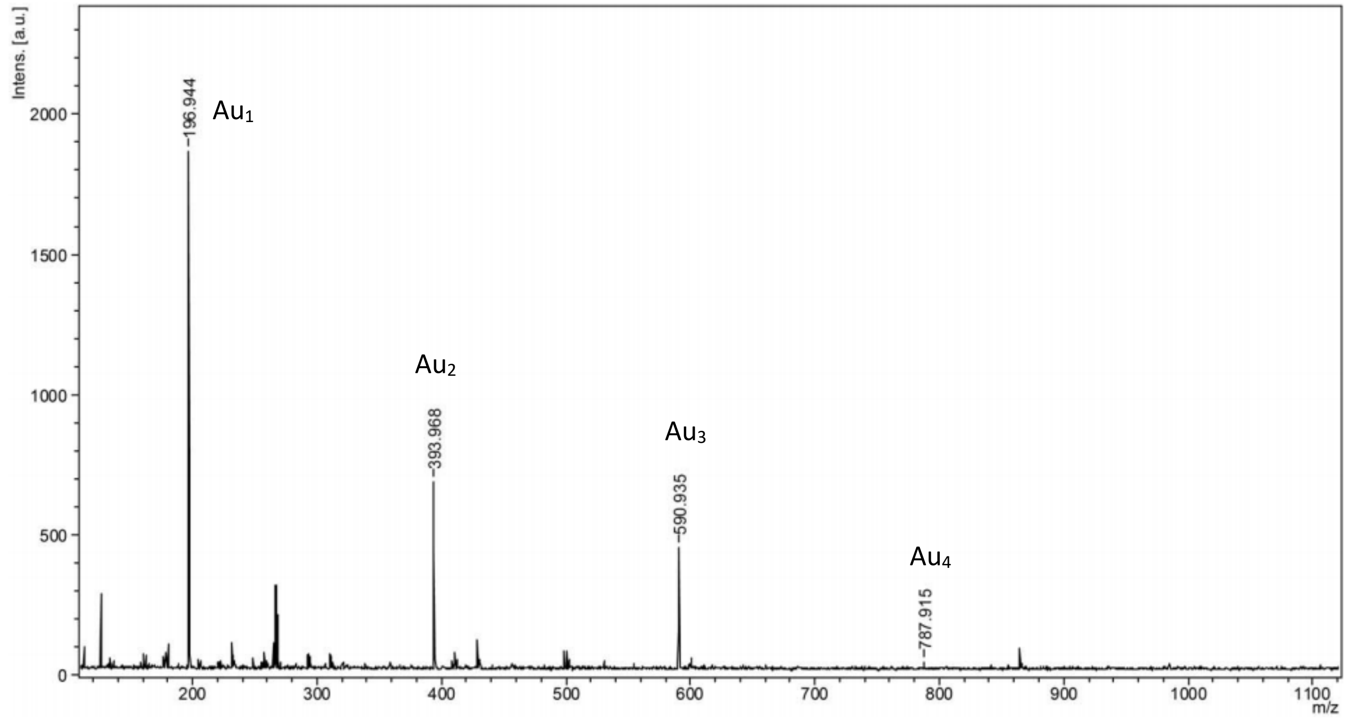

Figure S3. MALDI-TOF in its negative mode showing the formation of small gold clusters inside of a polyethylene glycol matrix (**5**).

a

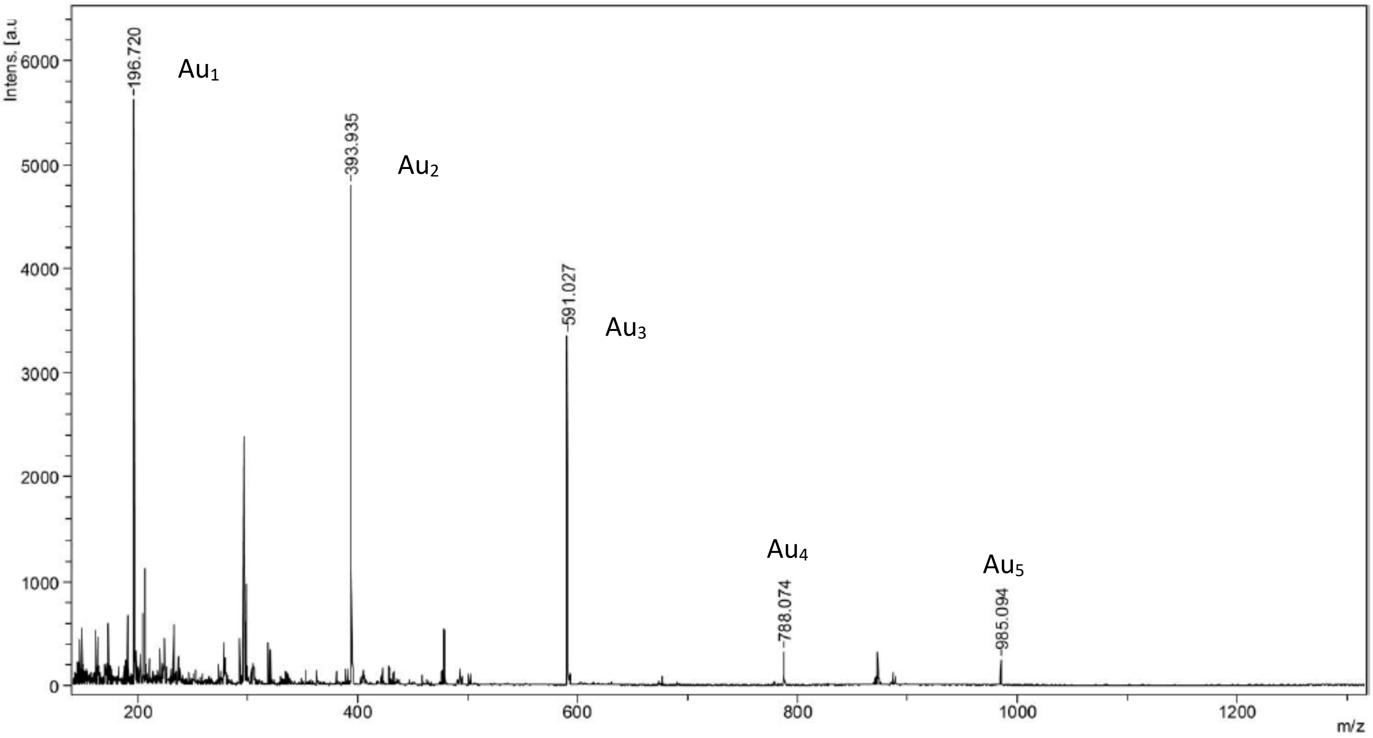

b

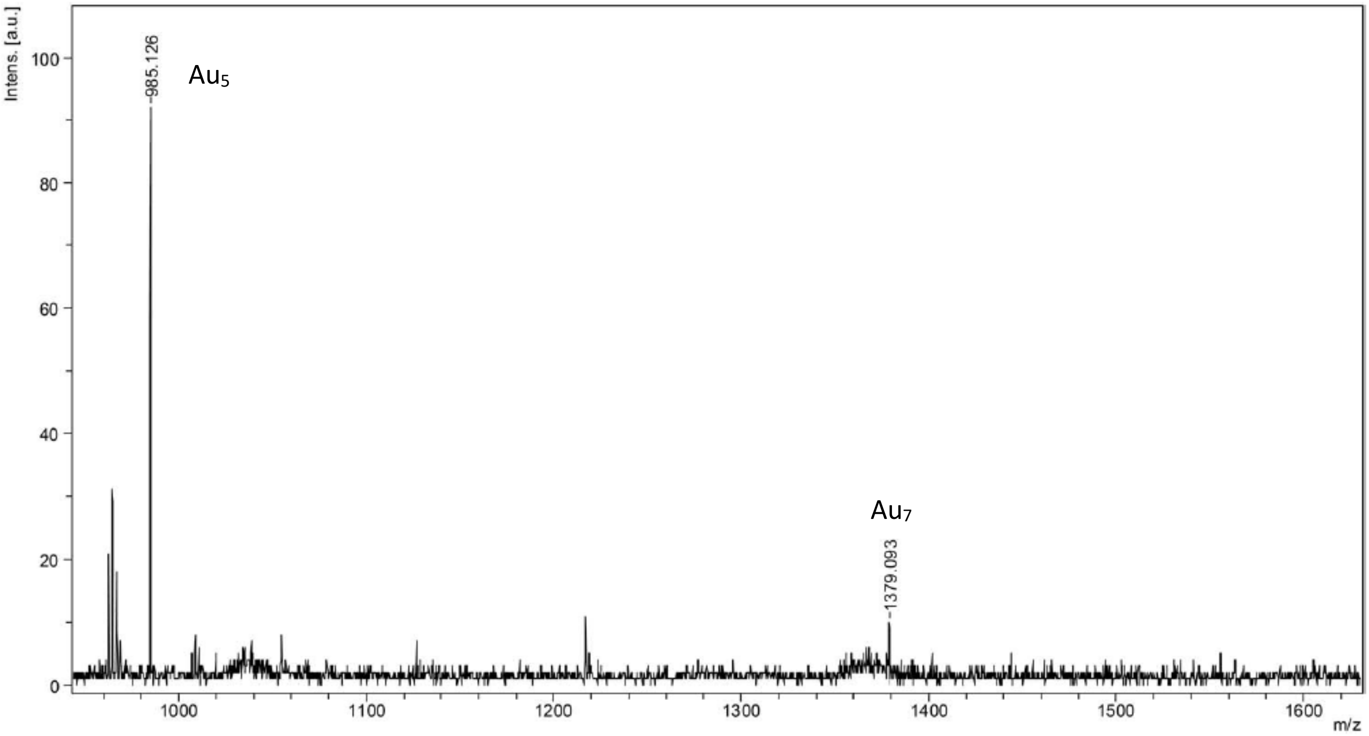

Figure S4. MALDI-TOF spectra in the **a,** positive and **b,** negative mode showing the formation of small gold clusters at 30 minutes of reaction conditions, using 1-hexene (**4**) as gold stabilizer.

a

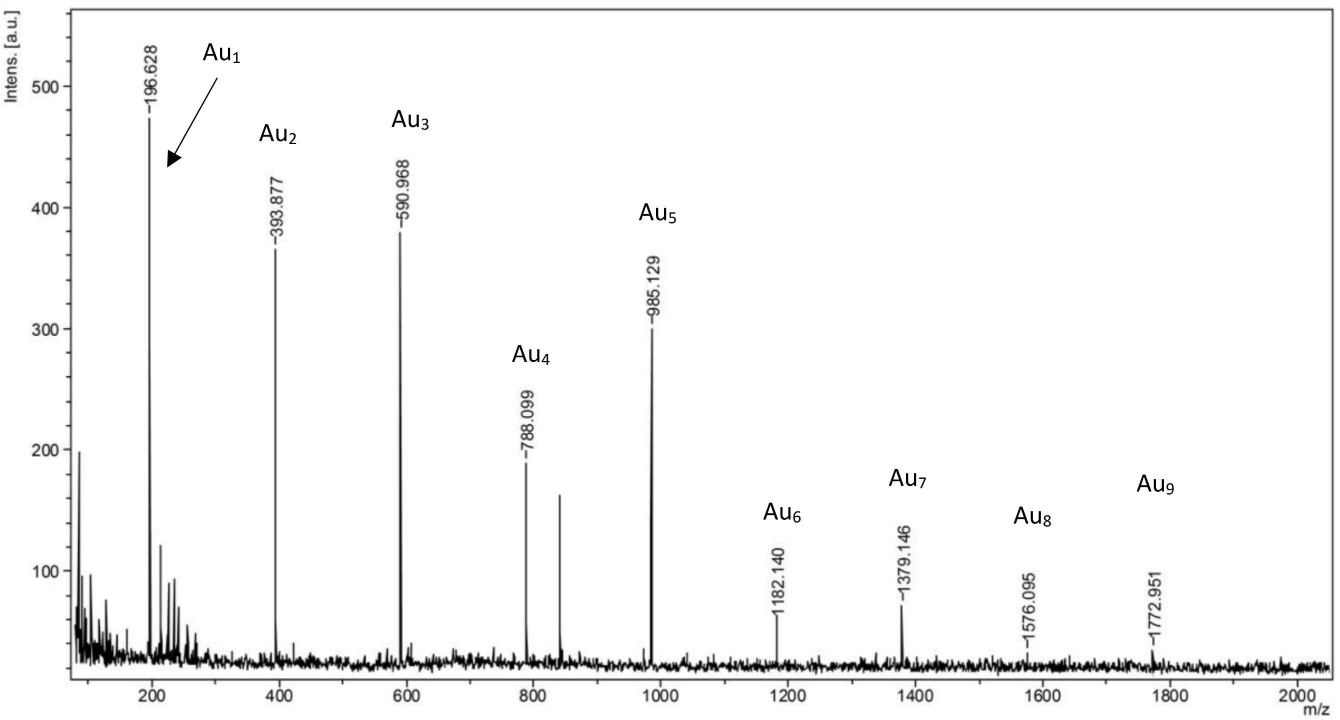

Figure S5. MALDI-TOF spectra showing the formation of small gold clusters at 30 minutes of reaction conditions, using 1-phenylpentane (**2**) as gold stabilizer.

a

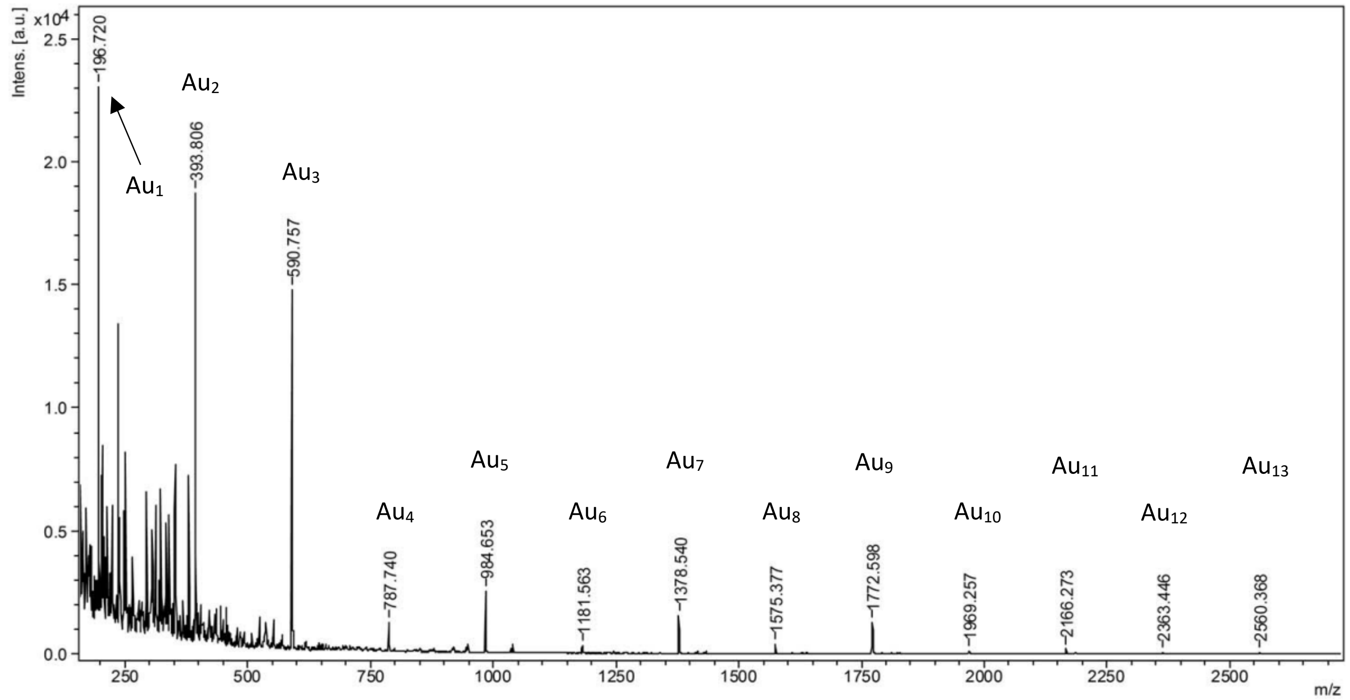

b

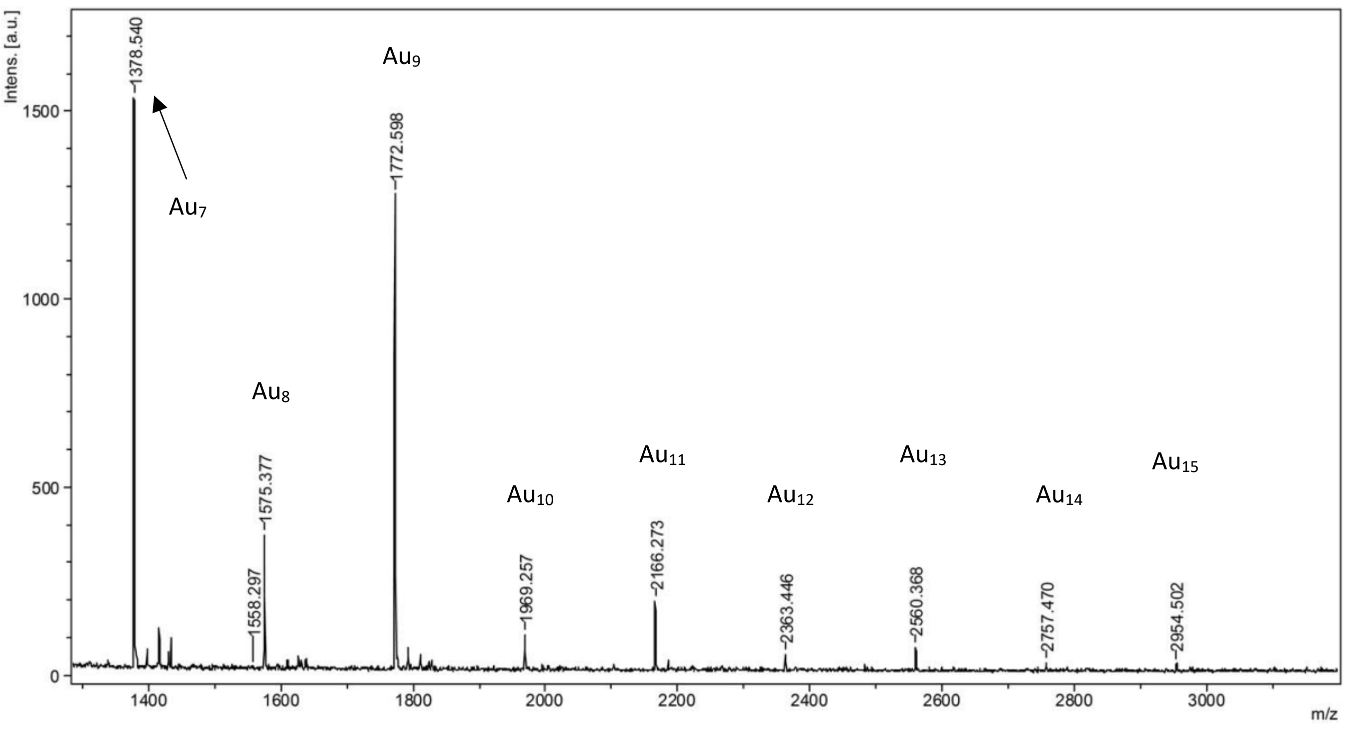

Figure S6. MALDI-TOF spectra in the **a,** 0-2600 m/z and **b,** 1250-3200 m/z ranges showing the formation of small gold clusters at 30 minutes of reaction conditions, using 5-phenylpentan-2-one (**3**) as gold stabilizer.

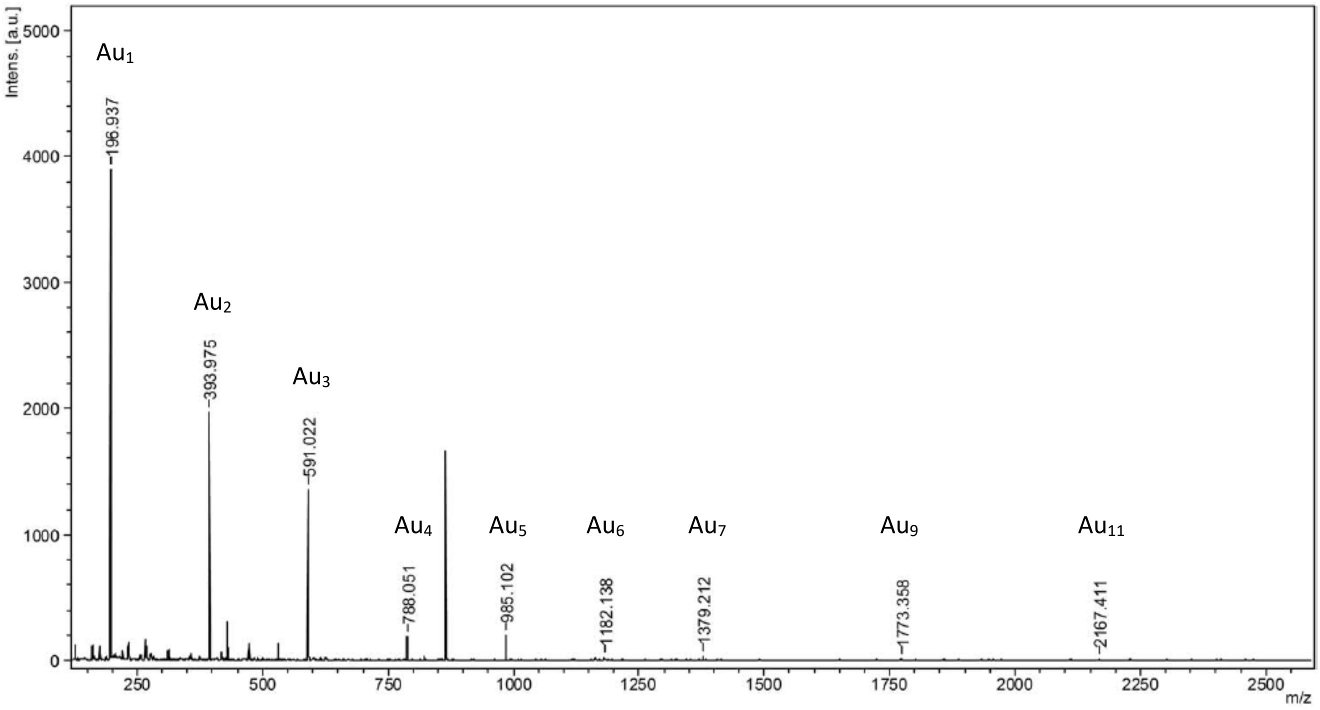

Figure S7. MALDI-TOF(-) spectra showing the formation of gold clusters in the hydration of 5-phenylpentyne (**1**) at 270 minutes of reaction conditions.

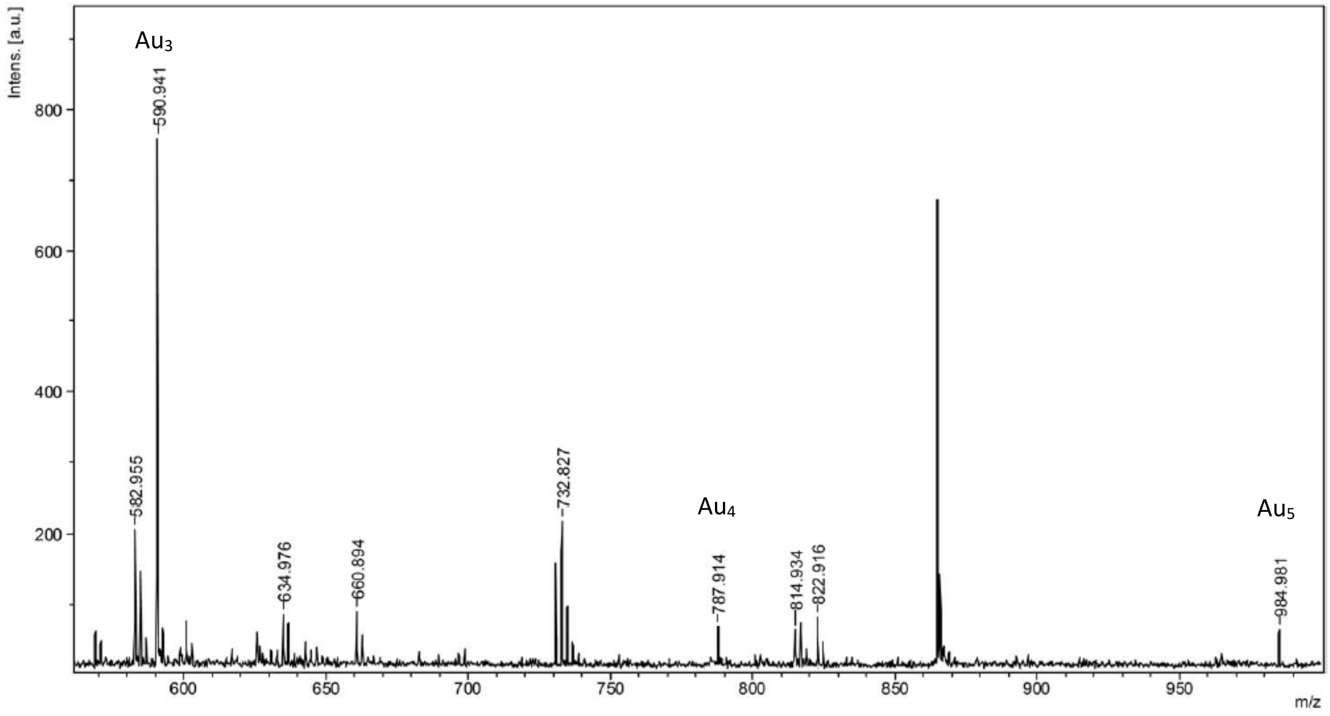

Figure S8. MALDI-TOF(-) spectra showing the formation of gold clusters in the hydration of 5-1-ethynylcyclohex-1-ene (**10**) at 30 minutes of reaction conditions.

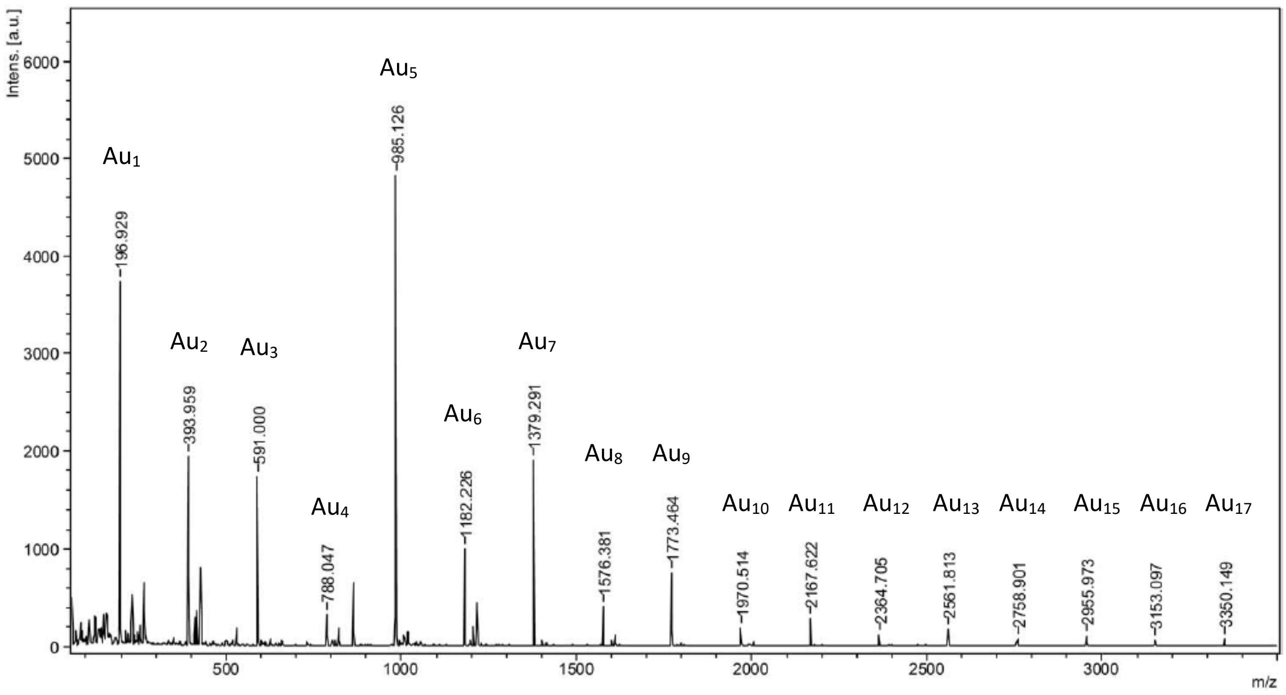

Figure S9. MALDI-TOF(-) spectra showing the formation of gold clusters in the hydration of 5-1-ethynylcyclohex-1-ene (**10**) at 60 minutes of reaction conditions.

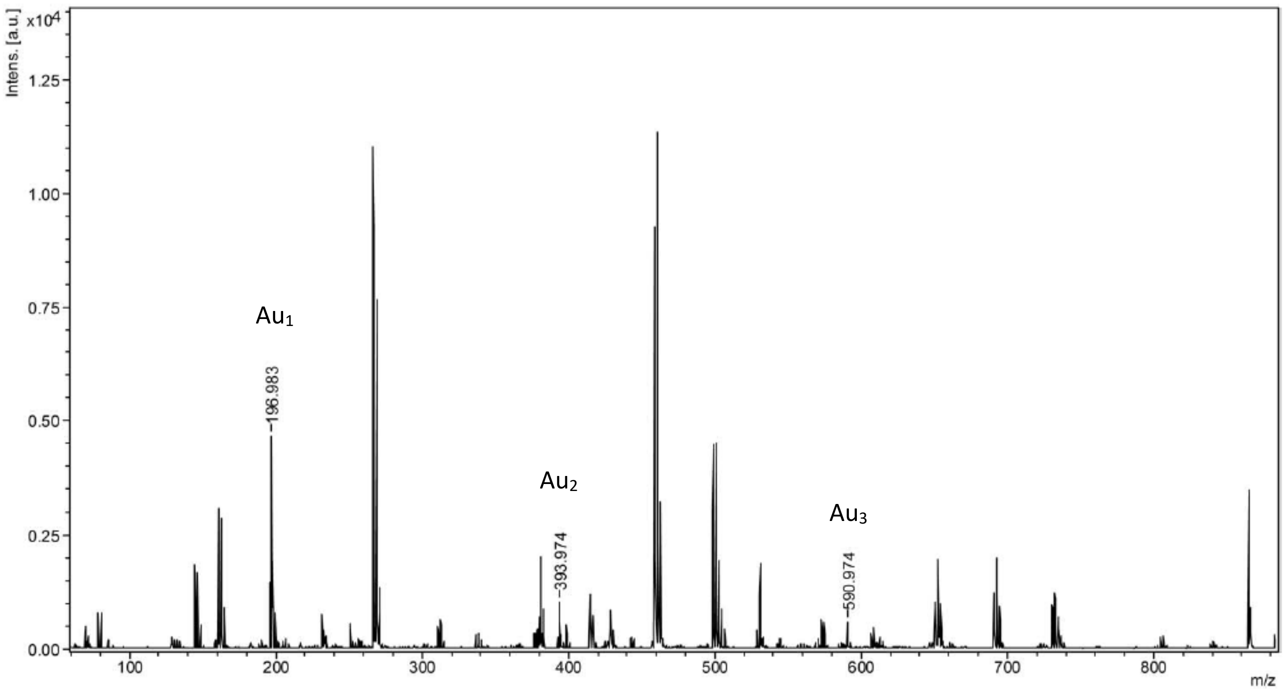

Figure S10. MALDI-TOF(-) spectra showing the formation of gold clusters in the hydration of 1-pentadecyne (**13**) at 30 minutes of reaction conditions.

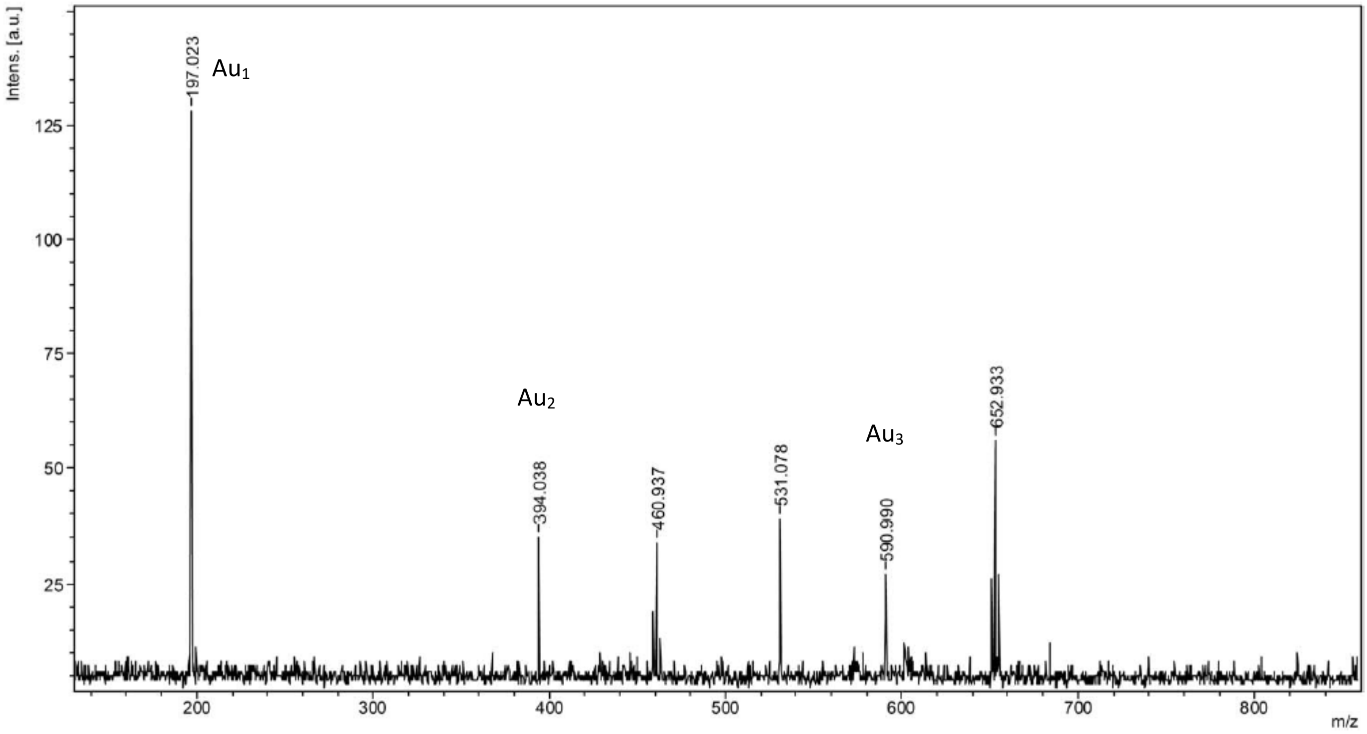

Figure S11. MALDI-TOF(-) spectra showing the formation of gold clusters in the hydration of 1-pentadecyne (**13**) at 210 minutes of reaction conditions.

a

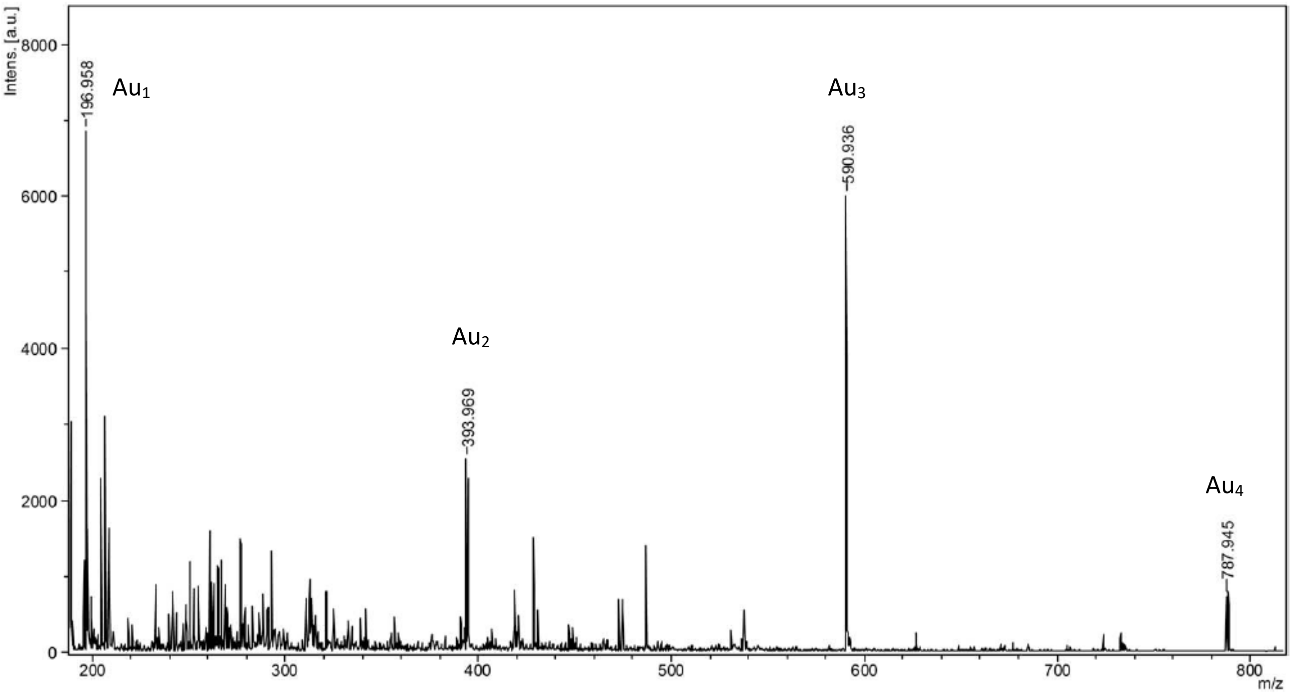

b

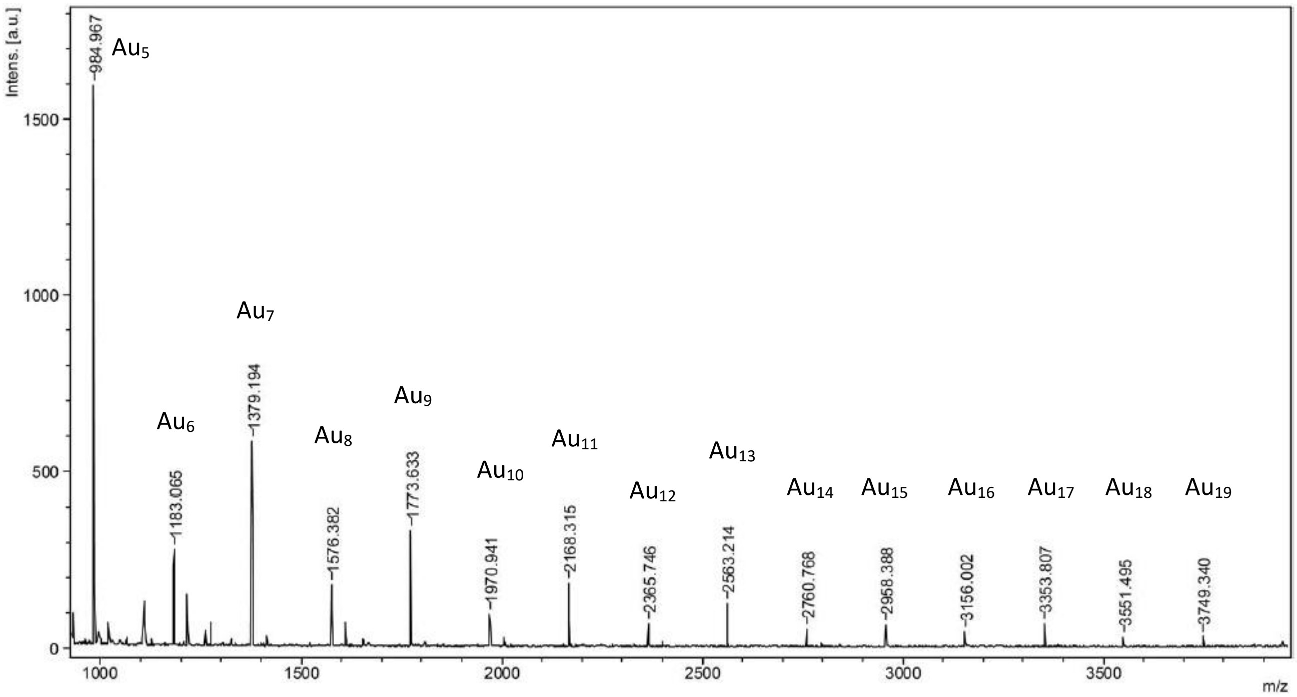

Figure S12. MALDI-TOF(-) spectra in the **a,** 0-900 m/z and **b,** 900-4000 m/z ranges showing the formation of gold clusters in the hydration of (prop-2-yn-1-yloxy)benzene (**11**) at 60 minutes of reaction conditions.

2 Computational Analysis

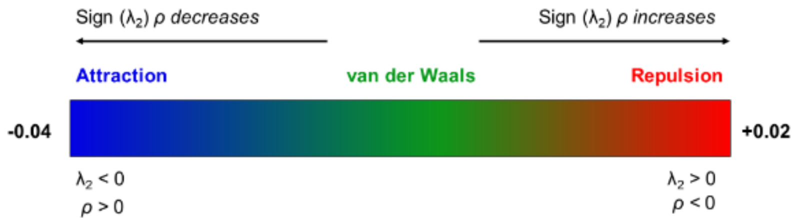

Figure S13. RGB colour scale refers to NCI and IRI surfaces and BCP QTAIM values.

2.1 Results and discussion

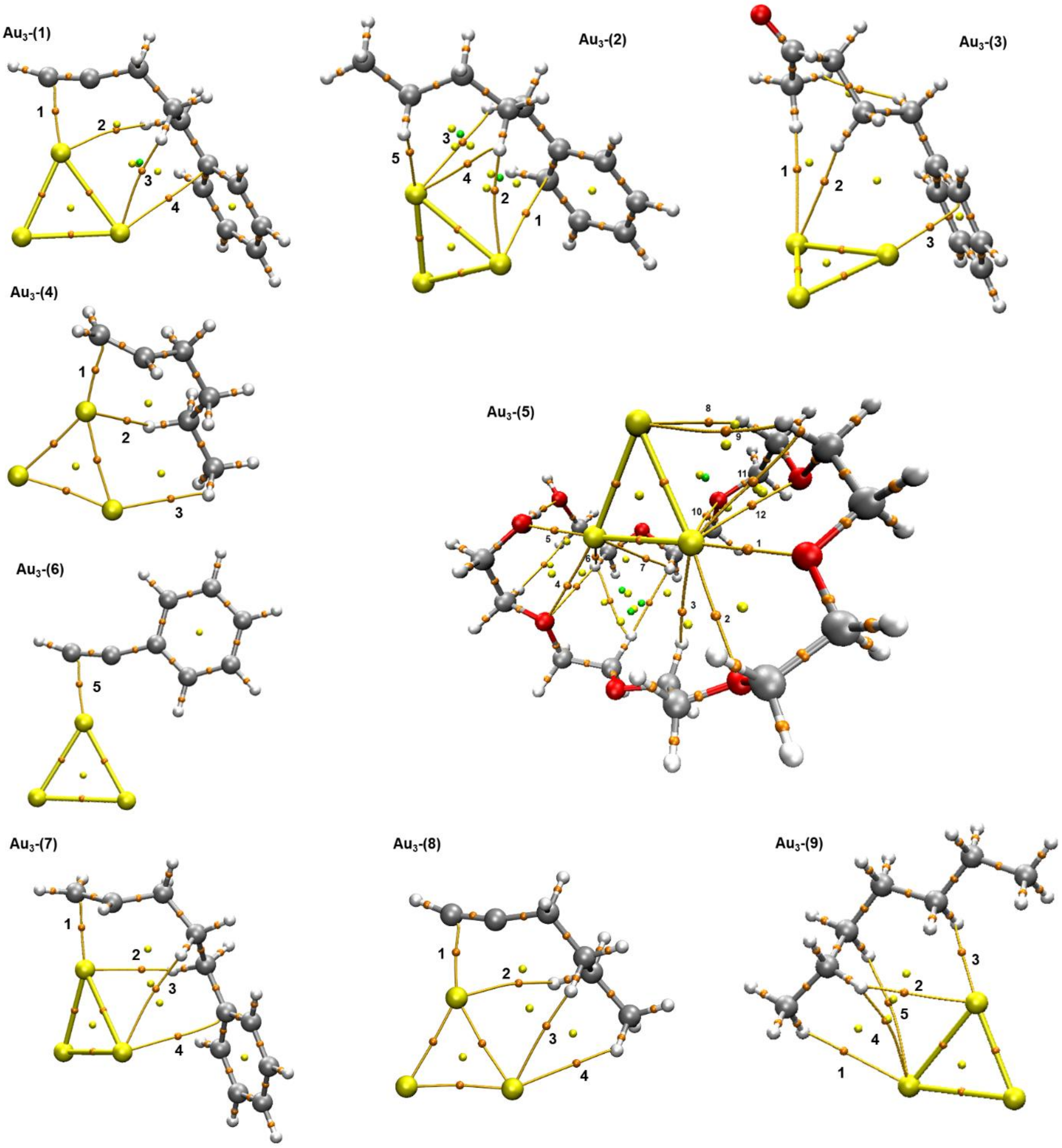

Figure S14. QTAIM analysis of the optimized Au_3-(**1-9**) theoretical models. Bond critical points are shown by orange balls, ring critical points by yellow balls and cage critical points by green balls.

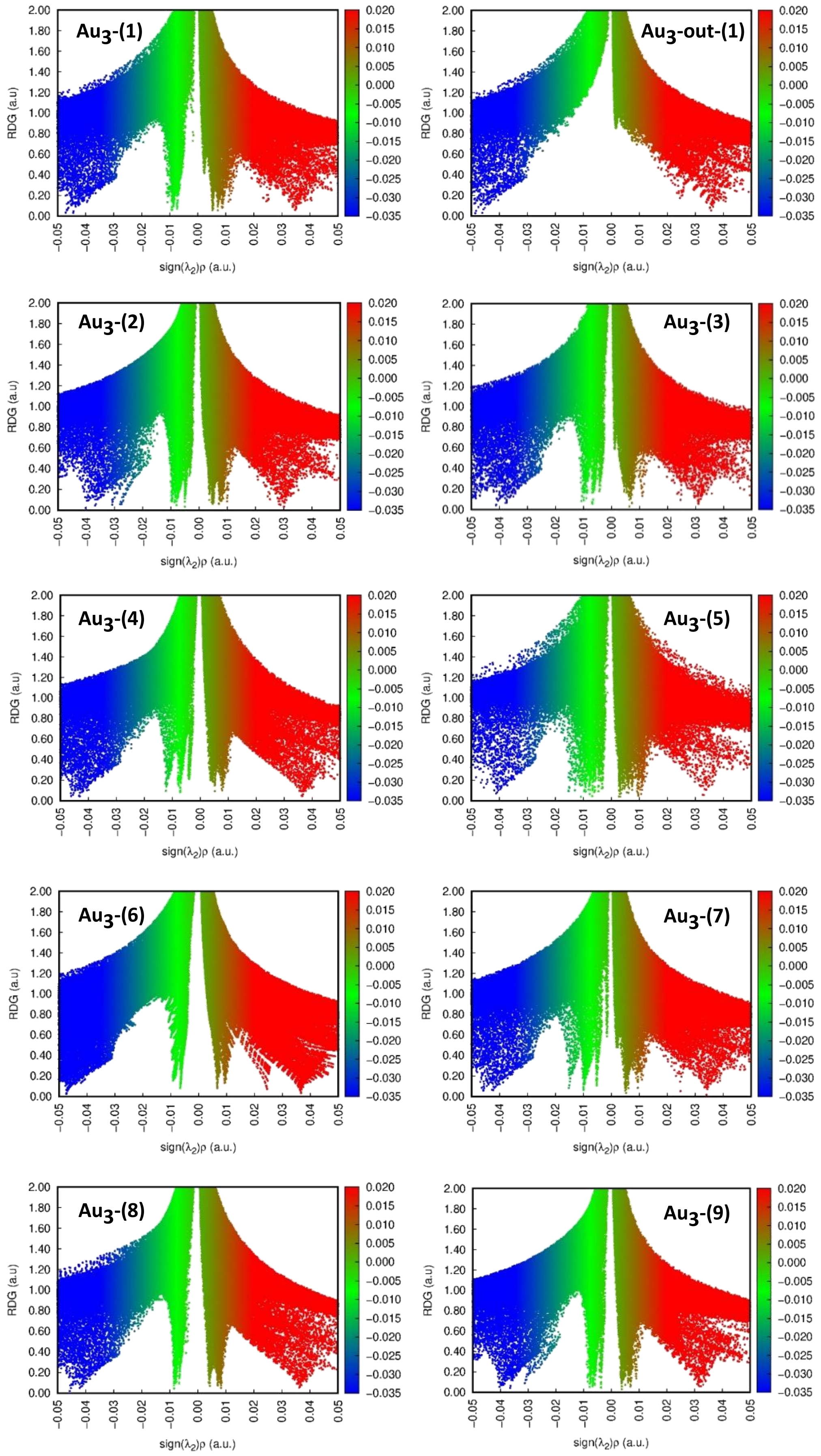

Figure S15. NCI scatter graphs of Au_3-(**1-9**) theoretical models.

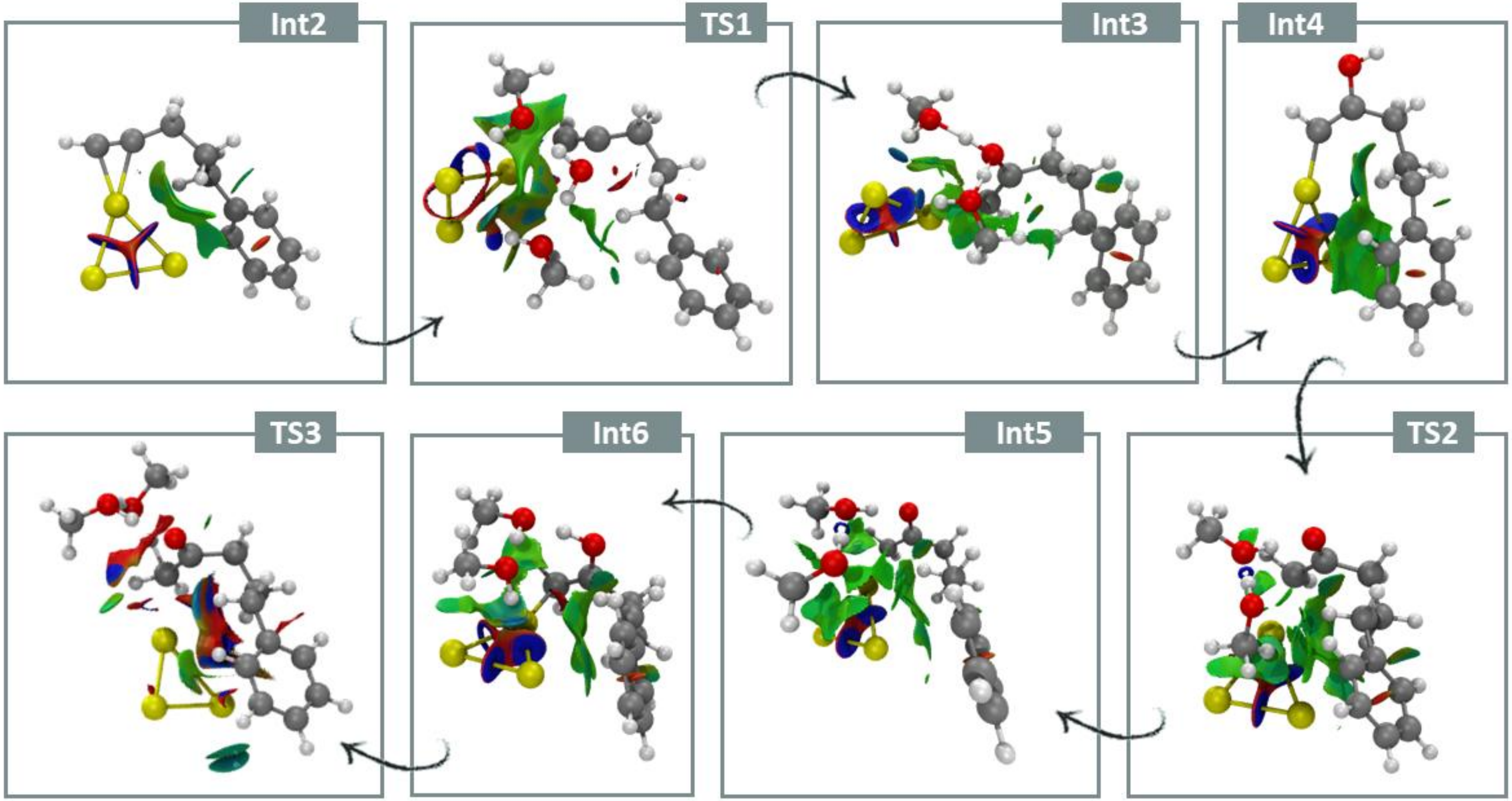

Figure S16. Minimum-energy theoretical models for the catalytic hydration of 5-phenylpentyne **(1)** calculated with M06-2X. NCI surfaces at different stages of the catalytic reaction. The isovalue is 0.4 a.u. and the color scale of -3 < ρ > 3

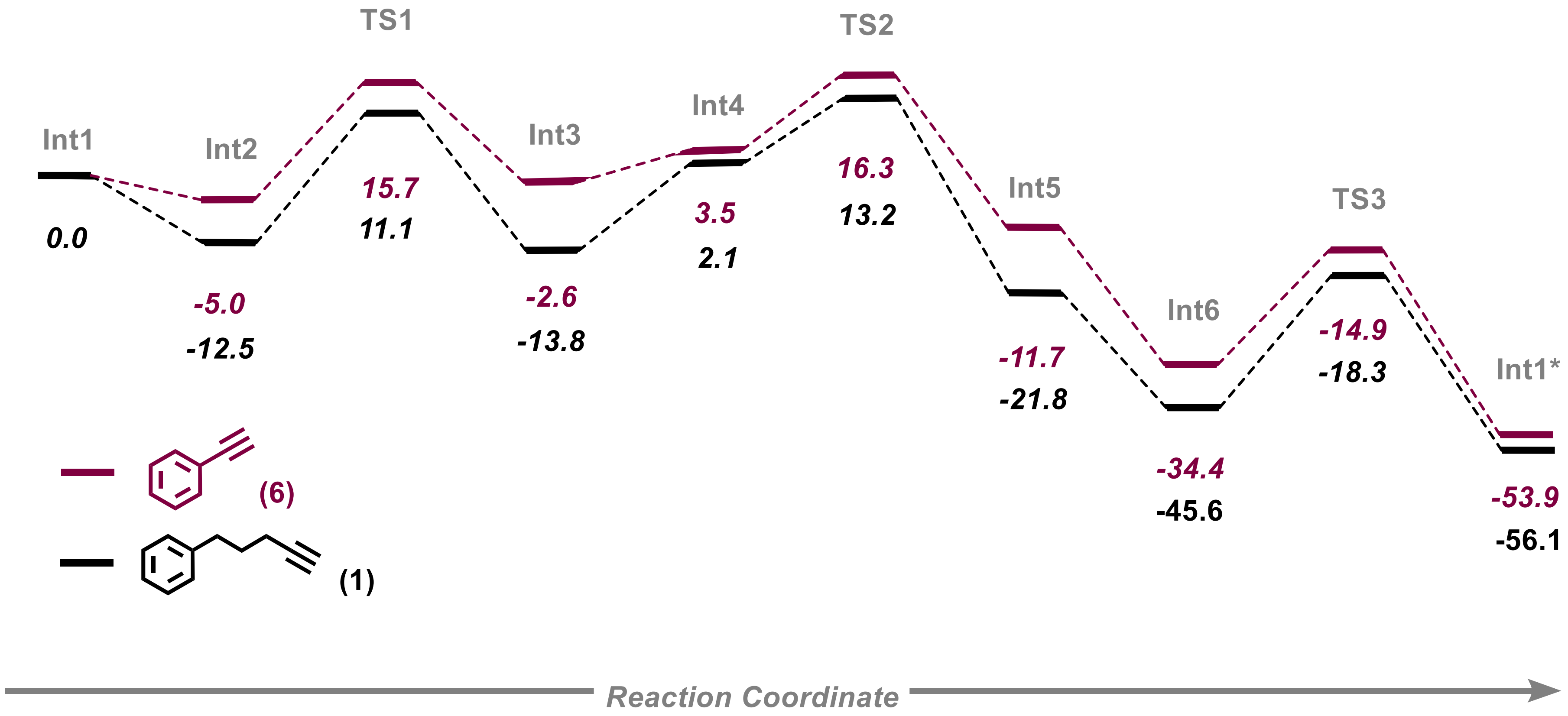

Figure S17. Minimum-energy reaction pathway comparison for the Au_3 catalyzed hydration of the 5-phenylpentyne **(1)** vs phenylacetylene **(6)** calculated with M06-2X. All the Gibbs free energies (ΔG) are given in kcal·mol^{-1}. Phenylacetylene data was reproduced from ref [Cordón, J.; Jiménez-Osés, G.; López-De-Luzuriaga, J. M.; Monge, M. The Key Role of Au-Substrate Interactions in Catalytic Gold Subnanoclusters. *Nat. Commun.* **2017**, *8* (1), 1–8. https://doi.org/10.1038/s41467-017-01675-1.]

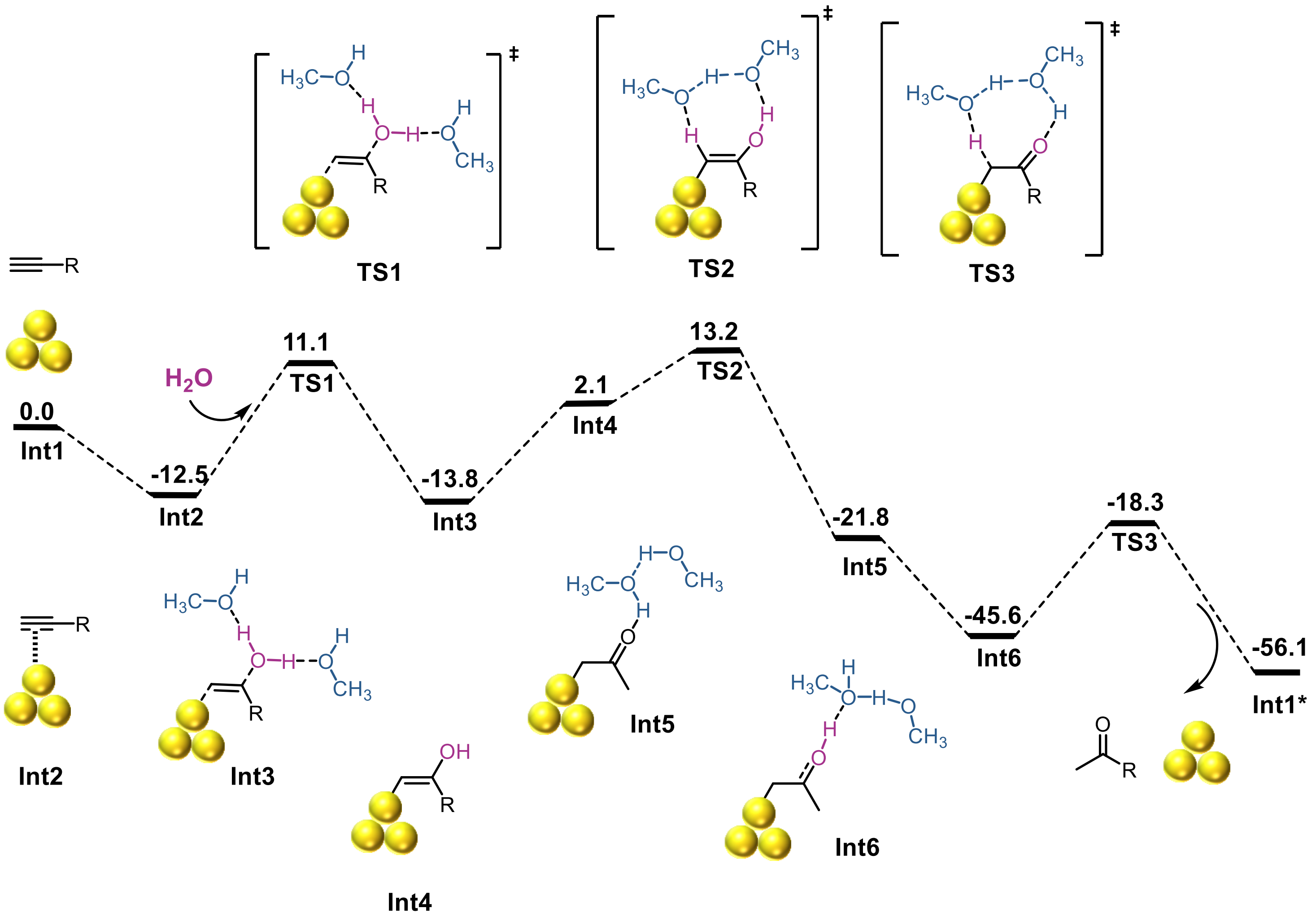

Figure S18. Minimum-energy reaction pathway for the Au_3 catalyzed hydration of the 5-phenylpentyne **(1)** calculated with M06-2X. All the Gibbs free energies (ΔG) are given in kcal·mol^{-1}.

Supplementary Tables

Table S1. Dissociation energies (E_{dis}) and interaction energies with the correction of counterpoisse (E_{int}) for the model systems (**1-9**) in kcal·mol^{-1}.

	E_{int}	E_{dis}
**Au_3-(1)**	-41.5	30.5
**Au_3_out-(1)**	-39.3	25.1
**Au_3-(2)**	-13.4	13.3
**Au_3-(3)**	-27.3	15.6
**Au_3-(4)**	-43.9	31.4
**Au_3-(5)**	-40.8	43.4
**Au_3-(6)**	-42.6	27.0
**Au_3-(7)**	-43.8	33.2
**Au_3-(8)**	-41.1	28.6
**Au_3_out-(8)**	-38.5	24.6
**Au_3-(9)**	-13.4	11.4

Table S2. Values of some chosen QTAIM parameters characterizing the model systems shown in Supplementary Figure S15.

	BCP	ρ_b (e·bohr^{-3})	$\nabla^2\rho_b$ (e·bohr^{-5})	V_b (e·bohr^{-3})	G_b (e· bohr^{-3})	E (e· bohr^{-3})	Interaction	Distances (Å)
**Au_3-(1)**	1	0.0932	0.198	-0.1073	0.0782	-0.0291	Au···π	2.192
	2	0.0088	0.026	-0.0046	0.0056	0.00097	Au···H-C(sp^3)	2.976
	3	0.0073	0.018	-0.0034	0.0040	0.00058	Au···H-C(sp^3)	3.087
	4	0.0092	0.023	-0.0047	0.0053	0.00054	Au···C_{ipso}(sp^2)	3.407
**Au_3-(2)**	1	0.0081	0.022	-0.0041	0.0047	0.00065	Au···C(sp^2)	3.528
	2	0.0075	0.018	-0.0034	0.0039	0.00054	Au···H-C(sp^3)	3.044
	3	0.0087	0.0258	-0.0050	0.0057	0.00070	Au···H-C(sp^3)	2.969
	4	0.0097	0.0273	-0.0055	0.0062	0.00064	Au···H-C(sp^3)	2.919
	5	0.0308	0.0927	-0.0251	0.0241	-0.00097	Au···H-C(sp^3)	2.309
**Au_3-(3)**	1	0.0063	0.014	-0.0027	0.0031	0.00039	Au···H-C(sp^3)	3.096
	2	0.0100	0.023	-0.0048	0.0053	0.00051	Au···H-C(sp^3)	2.854
	3	0.0525	0.132	-0.0470	0.0400	-0.00713	Au···C(sp^2)	2.480
**Au_3-(4)**	1	0.0917	0.1826	-0.1063	0.0758	-0.03048	Au···π	2.201
	2	0.0123	0.0362	-0.0082	0.0086	0.00044	Au···H-C(sp^3)	2.723
	3	0.0071	0.0184	-0.0038	0.0042	0.00041	Au···H-C(sp^3)	3.073
**Au_3-(5)**	1	0.04729	0.1941	-0.0559	0.0522	-0.00376	Au···O	2.397
	2	0.01536	0.05115	-0.0113	0.0120	0.00073	Au···O	3.037
	3	0.008120	0.02165	-0.0042	0.0048	0.00062	Au···H-C(sp^3)	3.014
	4	0.01321	0.0449	-0.0094	0.0103	0.00093	Au···O	3.066
	5	0.00783	0.02035	-0.0040	0.0046	0.00052	Au···O	2.305
	6	0.05987	0.2409	-0.0763	0.0683	-0.00802	Au···H-C(sp^3)	2.980
	7	0.00681	0.01751	-0.0032	0.0038	0.00059	Au···H-C(sp^3)	3.077
	8	0.01088	0.02533	-0.0053	0.0059	0.00048	Au···H-C(sp^3)	2.810
	9	0.00769	0.01802	-0.0034	0.0040	0.00053	Au···H-C(sp^3)	3.060
	10	0.004664	0.01514	-0.0022	0.0030	0.00081	Au···O	3.659
	11	0.009741	0.03267	-0.0065	0.0073	0.00084	Au···H-C(sp^3)	2.968
	12	0.01155	0.04019	-0.0079	0.0090	0.00108	Au···O	3.161
**Au_3-(6)**	1	0.0991	0.2218	-0.1197	0.0874	-0.03232	Au···π	2.165
**Au_3-(7)**	1	0.0907	0.176	-0.1000	0.0720	-0.02911	Au···π	2.216
	2	0.0152	0.0418	-0.0091	0.0098	0.00099	Au···H-C(sp^3)	2.628
	3	0.0053	0.0145	-0.0023	0.0030	0.00058	Au···H-C(sp^3)	3.288
	4	0.0100	0.0258	-0.0053	0.0059	0.00054	Au···C_{ipso}(sp^2)	3.380
**Au_3-(8)**	1	0.0916	0.2187	-0.1116	0.0830	-0.02866	Au···π	2.189
	2	0.0081	0.0202	-0.0050	0.0055	0.00056	Au···H-C(sp^3)	2.972
	3	0.0085	0.0244	-0.0043	0.0047	0.00035	Au···H-C(sp^3)	3.010
	4	0.0066	0.0161	-0.0034	0.0037	0.00031	Au···H-C(sp^3)	3.110
**Au_3-(9)**	1	0.0078	0.020	-0.0040	0.0045	0.00050	Au···H-C(sp^3)	2.997
	2	0.0076	0.020	-0.0039	0.0045	0.00017	Au···H-C(sp^3)	2.999
	3	0.0367	0.110	-0.0316	0.0295	-0.00214	Au···H-C(sp^3)	3.210
	4	0.0061	0.016	-0.0026	0.0033	0.00066	Au···H-C(sp^3)	3.221
	5	0.0037	0.010	-0.0014	0.0020	0.00058	Au···H-C(sp^3)	3.495

Table S3. Natural Energy Decomposition Analysis (NEDA) of the model systems Au_3-(**1-9**) in kcal·mol^{-1}.

Nombre	E_{ct}	E_{es}	E_{pol}	E_{xc}	E_{def}	E_{se}	E_{el}	E_{core}	E_{int}	% ionic	% CT	CORE-(EL+CT)
Au_3-(1)	-110,6	-76,74	-24,66	-54,44	232,53	13,80	-87,60	164,29	-33,92	28,80	41,51	-33,91
Au_3_out-(1)	-105,46	-72,00	-23,52	-46,55	211,48	13,09	-82,44	151,85	-36,05	29,09	42,60	-36,05
Au_3-(2)	-34,38	-16,73	-15,45	-24,56	86,45	7,90	-24,28	53,98	-4,68	18,36	37,73	-4,68
Au_3-(3)	-59,86	-38,00	-26,65	-36,84	141,99	14,77	-49,88	90,38	-19,36	23,55	37,10	-19,36
Au_3-(4)	-119,12	-81,35	-22,61	-53,29	238,20	13,28	-90,69	171,63	-38,17	29,43	43,10	-38,17
Au_3-(5)	-95,68	-68,51	-91,81	-71,54	302,14	47,08	-113,24	183,53	-25,39	20,92	29,21	-25,39
Au_3-(6)	-120,72	-76,34	-25,76	-50,88	235,24	14,29	-87,81	170,07	-38,46	27,89	44,10	-38,46
Au_3-(7)	-116,58	-81,46	-24,15	-57,50	244,29	13,90	-91,70	172,89	-35,39	29,13	41,68	-35,39
Au_3-(8)	-110,36	-75,35	-23,62	-50,15	223,72	13,40	-85,57	160,17	-35,76	29,04	42,53	-35,76
Au_3_out-(8)	-102,79	-71,22	-22,97	-45,86	207,57	12,83	-81,35	148,87	-35,27	29,33	42,33	-35,27
Au_3-(9)	-37,65	-17,30	-12,46	-20,39	80,26	6,51	-23,25	53,36	-7,55	19,70	42,88	-7,55

2.2 Cartesian coordinates of the lowest energy calculated structures

Au3-(1)

```
0 2
 C                   0.47661869    0.55755395    0.00000000
 C                  -0.62044631    0.51434695   -0.56724700
 H                   1.37560869    0.86397495    0.51480600
 Au                  1.50476069   -3.93060505   -0.38600000
 Au                 -1.22776731   -3.91023105   -1.00724700
 Au                  0.28967369   -1.57825205   -0.45644700
 C                  -1.91129731    0.79816795   -1.21130200
 H                  -2.38233531    1.61024195   -0.63620800
 H                  -1.69497331    1.20184995   -2.21060100
 C                  -2.86123331   -0.38993205   -1.32720800
 H                  -3.73167831   -0.06053105   -1.91161500
 H                  -2.36879531   -1.18734305   -1.90787200
 C                  -3.30211131   -0.93537205    0.02140300
 H                  -2.41882531   -1.07222505    0.66787000
 H                  -3.92884031   -0.18324505    0.52991900
 C                  -4.03859331   -2.25113005   -0.03176900
 C                  -4.65953831   -2.71625205   -1.19356100
 C                  -4.09004031   -3.05608905    1.11211500
 C                  -5.30223331   -3.95352505   -1.21539000
 H                  -4.63503531   -2.11686205   -2.10663800
 C                  -4.73053531   -4.29016505    1.09588600
 H                  -3.60205631   -2.71039505    2.02884700
 C                  -5.33677431   -4.74755705   -0.07343600
 H                  -5.77540331   -4.29961505   -2.13780000
 H                  -4.75048331   -4.90362405    2.00021800
 H                  -5.83550931   -5.71955405   -0.09214500
```

Au3_out-(1)

```
0 2
 Au                 -0.92523363    0.59813086    0.00000000
 Au                 -2.46530463   -1.75281414    0.06283100
 Au                  0.18569237   -1.95274214   -0.03359700
 C                   1.91173037   -3.31559914   -0.09923900
 H                   1.73492437   -4.38074414   -0.12607100
 C                   2.40536437   -2.18163614   -0.07606100
 C                   3.28374237   -1.00762314   -0.05187100
 H                   3.06368737   -0.38375414   -0.93128100
 H                   3.03538537   -0.39905114    0.83058600
 C                   4.75858637   -1.40574614   -0.03032100
 H                   4.95337637   -2.03995914    0.84793400
 H                   4.98702337   -2.01451414   -0.91838900
 C                   5.67209437   -0.18378714    0.00550200
 H                   5.43134837    0.42039286    0.89334100
 H                   5.46991737    0.44466486   -0.87498000
 C                   7.12601437   -0.57044714    0.03230200
 C                   7.83981237   -0.75134014   -1.15563300
 C                   7.77936437   -0.80748714    1.24507000
 C                   9.17201237   -1.15569714   -1.13407100
 H                   7.34243737   -0.56731614   -2.11316800
 C                   9.11128637   -1.21205214    1.27183200
 H                   7.23417737   -0.66783314    2.18394100
 C                   9.81218637   -1.38763914    0.08099100
```

H	9.71507937	-1.28744814	-2.07338700
H	9.60642837	-1.38836714	2.23014000
H	10.85838437	-1.70257114	0.10002200

(1)

0 1

C	1.00759900	0.00003500	0.51504300
C	1.65230800	1.20080900	0.20443600
C	1.65228600	-1.20078200	0.20457000
C	2.90775500	1.20355400	-0.39753500
H	1.16143300	2.14965100	0.44310100
C	2.90773500	-1.20361800	-0.39740200
H	1.16139900	-2.14959200	0.44333900
C	3.53994000	-0.00005600	-0.70058900
H	3.39751700	2.15298800	-0.62859400
H	3.39747400	-2.15309000	-0.62835300
H	4.52651900	-0.00008500	-1.17046000
C	-0.37112500	0.00008400	1.11708200
H	-0.49507000	0.88432900	1.76092200
H	-0.49507600	-0.88406000	1.76105800
C	-1.46406700	0.00000500	0.05222300
H	-1.34823400	0.88259100	-0.59590000
H	-1.34824000	-0.88268100	-0.59576300
C	-2.86166200	0.00005900	0.66871000
H	-2.98047600	0.88082700	1.31872600
H	-2.98048000	-0.88060100	1.31887100
C	-3.92513700	-0.00002000	-0.33183200
C	-4.78579300	-0.00009200	-1.18326000
H	-5.55579600	-0.00014700	-1.93562400

Au3-(2)

0 2

Au	1.84551900	1.21339500	0.49719900
Au	0.80926800	-0.84024100	-1.23991900
Au	-0.72099300	1.24163200	0.14244300
C	-4.10879700	-0.16816600	-0.35475200
H	-4.89586300	-0.36134000	0.39338400
H	-4.60897400	-0.16126900	-1.33538000
C	-3.07942900	-1.29103200	-0.31828400
H	-3.50915800	-2.19388200	-0.77632000
H	-2.21620400	-1.01478200	-0.95238600
C	-2.58777400	-1.62418200	1.09119900
H	-2.38365400	-0.69928600	1.65333500
H	-3.40081600	-2.13285300	1.63586700
C	-1.34211800	-2.47206900	1.12110200
C	-1.24469700	-3.64594800	0.36534900
C	-0.23929600	-2.08923100	1.88919500
C	-0.07828000	-4.40592400	0.37005400
H	-2.09491200	-3.97246800	-0.24062500
C	0.93057000	-2.84562900	1.89843300
H	-0.29385100	-1.16775100	2.47788900
C	1.01693600	-4.00613000	1.13430200
H	-0.02350100	-5.31832000	-0.22932300
H	1.78246900	-2.51988000	2.50122400
H	1.93394800	-4.60044300	1.13534800

C	-3.52281400	1.21704600	-0.11029000
H	-3.02370900	1.23798500	0.87883100
H	-2.77077000	1.41381300	-0.90749400
C	-4.54736700	2.33926000	-0.14479400
H	-4.07750900	3.31775600	0.02077300
H	-5.06039500	2.36637800	-1.11662800
H	-5.30841600	2.19028400	0.63430100

(2)

0 1

C	-3.51764100	1.71631900	-0.14711400
H	-3.45667200	2.09195700	0.88325600
H	-4.55185600	1.74655300	-0.50568600
H	-2.89773600	2.38073500	-0.76778300
C	-2.96847000	0.32407100	-0.23750800
C	-1.64710400	0.07024700	0.43380600
H	-0.94344000	0.84386200	0.08153600
H	-1.78400100	0.28655200	1.50797300
C	-1.09262800	-1.32389500	0.22625600
H	-0.97144000	-1.51162300	-0.85226600
H	-1.82426500	-2.06233400	0.58468200
C	0.24184900	-1.54731300	0.93867400
H	0.11057700	-1.38160300	2.01880800
H	0.53580300	-2.59933400	0.81072200
C	1.34371900	-0.65730800	0.42574200
C	1.94788700	-0.92251700	-0.80802500
C	1.76282900	0.46797100	1.14012100
C	2.94230000	-0.09013700	-1.31226200
H	1.63529400	-1.80248800	-1.37962400
C	2.75780700	1.30552800	0.63971900
H	1.30174900	0.68978300	2.10771600
C	3.35092600	1.02885300	-0.58874800
H	3.40531300	-0.31805700	-2.27578700
H	3.07219600	2.17948400	1.21596300
H	4.13301200	1.68284900	-0.98210200
O	-3.56167200	-0.55715700	-0.83892100

Au3-(3)

0 2

Au	-0.40467624	0.48561150	0.00000000
Au	-1.60384124	-2.11260950	-0.41973500
Au	-2.98856824	0.40422150	-0.20166100
C	-5.56917424	2.85493050	-0.11858400
H	-5.00285624	2.77786150	0.82287500
H	-6.12935524	3.79934950	-0.09688600
H	-4.83866324	2.91749150	-0.94087000
C	-6.48762324	1.65910450	-0.29838000
H	-7.23365424	1.63017050	0.51113700
H	-7.05356624	1.75400950	-1.23830200
C	-5.73017124	0.34051150	-0.30948900
H	-5.19501024	0.24634350	0.65768000
H	-4.99740724	0.38132250	-1.15154500
C	-6.58440024	-0.90015050	-0.52499500
H	-7.11932724	-0.78904450	-1.48087100
H	-7.35771724	-0.93726950	0.26032000
C	-5.80359424	-2.20854650	-0.52730500
H	-6.47178524	-3.01765050	-0.86332300

H	-4.99192924	-2.15273750	-1.27722600
C	-5.20789424	-2.59877550	0.81922700
H	-4.42710524	-1.87532950	1.11241600
H	-5.99105024	-2.53632750	1.59336600
C	-4.60839224	-3.99499450	0.80478200
H	-4.15214424	-4.25163650	1.77138500
H	-5.37542924	-4.75117450	0.57977600
H	-3.82389424	-4.07420350	0.03450500

(3)

0 1

C	3.80767700	-0.35019900	-0.00000100
H	4.71254200	0.27322400	-0.00000400
H	3.84754400	-1.00005000	0.88703100
H	3.84754100	-1.00005200	-0.88703200
C	2.54328800	0.49202200	0.00000000
H	2.53796300	1.15654500	0.87970200
H	2.53796100	1.15654400	-0.87970300
C	1.27166100	-0.34171100	0.00000200
H	1.27223400	-1.00739400	0.88057700
H	1.27223400	-1.00739700	-0.88057200
C	0.00000000	0.49060500	0.00000100
H	0.00000000	1.15609500	-0.88045600
H	0.00000000	1.15609600	0.88045700
C	-1.27166100	-0.34171100	0.00000000
H	-1.27223400	-1.00739500	0.88057500
H	-1.27223300	-1.00739600	-0.88057400
C	-2.54328800	0.49202200	0.00000000
H	-2.53796100	1.15654400	-0.87970300
H	-2.53796200	1.15654500	0.87970200
C	-3.80767700	-0.35019900	-0.00000100
H	-3.84754300	-1.00005100	0.88703100
H	-4.71254200	0.27322400	-0.00000100
H	-3.84754200	-1.00005100	-0.88703200

Au3-(4)

0 2

Au	-0.89028773	0.46758582	0.00612103
Au	-1.92653873	-1.58605018	-1.73099697
Au	-3.45679973	0.49582282	-0.34863497
C	-6.84460373	-0.91397518	-0.84582997
H	-7.63166973	-1.10714918	-0.09769397
H	-7.34478073	-0.90707818	-1.82645797
C	-5.81523573	-2.03684118	-0.80936197
H	-6.24496473	-2.93969118	-1.26739797
H	-4.95201073	-1.76059118	-1.44346397
C	-5.32358073	-2.36999118	0.60012103
H	-5.11946073	-1.44509518	1.16225703
H	-6.13662273	-2.87866218	1.14478903
C	-4.07792473	-3.21787818	0.63002403
C	-3.98050373	-4.39175718	-0.12572897
C	-2.97510273	-2.83504018	1.39811703
C	-2.81408673	-5.15173318	-0.12102397
H	-4.83071873	-4.71827718	-0.73170297
C	-1.80523673	-3.59143818	1.40735503
H	-3.02965773	-1.91356018	1.98681103

C	-1.71887073	-4.75193918	0.64322403
H	-2.75930773	-6.06412918	-0.72040097
H	-0.95333773	-3.26568918	2.01014603
H	-0.80185873	-5.34625218	0.64427003
C	-6.25862073	0.47123682	-0.60136797
H	-5.75951573	0.49217582	0.38775303
H	-5.50657673	0.66800382	-1.39857197
C	-7.28317373	1.59345082	-0.63587197
H	-6.81331573	2.57194682	-0.47030497
H	-7.79620173	1.62056882	-1.60770597
H	-8.04422273	1.44447482	0.14322303

(4)

0 1

C	-2.62516600	-0.65221600	-0.50560900
H	-2.77069900	-1.55169800	0.11751800
H	-3.08293600	-0.88263700	-1.48157600
C	-1.13441000	-0.41597600	-0.70820600
H	-0.69698600	-1.27696500	-1.23822700
H	-0.98389000	0.45695500	-1.36607300
C	-0.35419100	-0.19734400	0.58829000
H	-0.72681700	0.70201200	1.10043400
H	-0.53485900	-1.04876900	1.26372400
C	1.12288800	-0.05009300	0.34381400
C	1.95991900	-1.16950600	0.31146800
C	1.68408000	1.20479400	0.08935800
C	3.31890800	-1.04027900	0.03748000
H	1.53696800	-2.15955600	0.50975300
C	3.04227900	1.34007000	-0.18597300
H	1.04289500	2.09180100	0.11225600
C	3.86514400	0.21652600	-0.21259400
H	3.95697100	-1.92768100	0.02221200
H	3.46191200	2.33099200	-0.37783500
H	4.93198100	0.32050500	-0.42524400
C	-3.37286400	0.51593900	0.12167900
H	-2.98489500	0.70872200	1.13438800
H	-3.16812500	1.42965400	-0.46162700
C	-4.87179600	0.27761000	0.19552200
H	-5.39648000	1.12575400	0.65669800
H	-5.29625300	0.12410000	-0.80790100
H	-5.09753200	-0.62033200	0.79012600

Au3-(5)

0 2

C	-1.32194239	1.65467623	0.00000000
H	-1.06606639	1.77379823	1.06426900
H	-1.44229339	2.67072823	-0.40745800
C	-2.64017939	0.88757523	-0.14995700
H	-3.47740539	1.59901823	-0.17960900
H	-2.64625139	0.36089023	-1.11970700
C	-2.86131939	-0.11233277	0.97927300
H	-1.91628639	-0.63341377	1.20881000
H	-3.11384439	0.44225023	1.89885300
C	-3.91088739	-1.16400577	0.72557600
C	-4.93628839	-0.98868277	-0.20707500
C	-3.85673039	-2.37356677	1.42852900

C	-5.87303439	-1.99557077	-0.43808500
H	-5.00549939	-0.05745677	-0.77491000
C	-4.78942639	-3.38002377	1.20330800
H	-3.05218339	-2.53162877	2.15405900
C	-5.80178739	-3.19592377	0.26231100
H	-6.66376739	-1.83840577	-1.17614700
H	-4.72093839	-4.31881577	1.75888400
H	-6.53309139	-3.98645977	0.07727600
C	1.09934361	0.86873623	-0.21345300
H	1.29742661	1.09377323	0.83670800
H	1.96342361	0.73673223	-0.86627600
C	-0.15733539	1.06197123	-0.74164000
H	-0.26630139	1.02335923	-1.83087200
Au	0.17131961	-1.14115777	-0.30450600
Au	0.22986361	-3.73001177	0.23567000
Au	-1.86785739	-2.82155477	-1.39691400

(5)

0 1

C	4.37423600	-0.87915400	0.33018400
H	4.53119200	-1.18106600	-0.71109600
H	5.23027200	-0.95317600	1.00610700
C	3.18835200	-0.44526900	0.75169300
H	3.07487700	-0.14850100	1.80299400
C	1.96716600	-0.30007800	-0.10017000
H	1.16659300	-0.94493900	0.30059500
H	2.18024900	-0.65726100	-1.12104600
C	1.46045000	1.13804400	-0.15120100
H	2.25791500	1.78826900	-0.54323100
H	1.25020400	1.48856300	0.87343500
C	0.20753700	1.31337900	-1.01091600
H	-0.03557100	2.38486400	-1.06554000
H	0.42483900	0.98238600	-2.03786200
C	-0.98487900	0.55927600	-0.48444400
C	-1.39447800	-0.64616200	-1.06021900
C	-1.68965200	1.03629400	0.62611600
C	-2.47640400	-1.35665300	-0.54447600
H	-0.85500600	-1.03369700	-1.93009900
C	-2.77150600	0.33154100	1.14507400
H	-1.38596600	1.98172100	1.08746100
C	-3.16879700	-0.86973800	0.56062300
H	-2.78088700	-2.29692300	-1.01128000
H	-3.31166000	0.72456800	2.01027100
H	-4.01920300	-1.42368800	0.96570000

Au3-(6)

0 2

C	-0.20681680	0.48561150	0.00270738
C	-0.90403480	1.51580250	0.00273538
H	0.13863720	-0.53839350	0.00267738
C	-2.03033380	2.41969350	0.00277038
C	-1.86617680	3.80975550	0.00277138
C	-3.32077180	1.86940550	0.00280738
C	-2.98253280	4.63793150	0.00280738
H	-0.85711680	4.23265550	0.00274338
C	-4.42914680	2.70589650	0.00284438

H	-3.44408080	0.78428350	0.00280638
C	-4.26269280	4.08973550	0.00284438
H	-2.84966680	5.72196350	0.00280838
H	-5.43208980	2.27349150	0.00287338
H	-5.13727580	4.74424150	0.00287438
Au	1.21794620	2.11544450	0.00274538
Au	3.89462620	2.38160050	0.00281238
Au	2.25504220	4.64536750	0.00275638

(6)

0 1

C	-3.23424500	0.00000000	-0.00000400
H	-4.31103200	-0.00000400	-0.00001200
C	-0.58681100	0.00000200	0.00001000
C	0.12077600	1.21043300	0.00000000
C	0.12077700	-1.21043000	0.00000000
C	1.51051400	1.20613300	-0.00000200
H	-0.43009500	2.15359800	-0.00000200
C	1.51051400	-1.20613600	-0.00000200
H	-0.43010000	-2.15359200	-0.00000200
C	2.20796500	-0.00000200	0.00000000
H	2.05407100	2.15372000	-0.00000500
H	2.05406200	-2.15372900	-0.00000500
H	3.30044300	0.00000600	-0.00000400
C	-2.02238100	0.00000000	0.00000200

Au3-(7)

0 2

Au	-0.37999400	-1.56030700	0.54009200
Au	-1.95863000	0.52111000	-0.45849200
Au	0.56972100	1.05530700	0.19109200
C	3.70711500	0.79844400	-0.19713300
H	3.66532300	1.25963800	-1.19636400
H	4.69749900	1.04802100	0.21671900
C	3.57322600	-0.72383000	-0.30858800
H	4.57466300	-1.17635300	-0.36916500
H	3.11389200	-1.12209500	0.61374300
C	2.76565500	-1.18187800	-1.51526900
H	1.77633100	-0.69133000	-1.51433400
H	3.27304200	-0.84025300	-2.43212000
C	2.06590900	2.65239800	0.42643400
H	2.19770400	3.13321500	-0.54528500
H	1.63382400	3.25655600	1.22541300
C	2.71963700	1.47099900	0.71239200
H	2.74077100	1.13904400	1.75607000
C	2.58064200	-2.68837400	-1.55457300
H	2.01309500	-3.00450300	-2.44103500
H	3.55184800	-3.20557800	-1.57216500
H	2.03221800	-3.03564500	-0.66374900

(7)

0 1

**C**	**0.86714700**	**0.99618600**	**0.28175900**
**H**	**0.97700800**	**0.84124100**	**1.36722900**
**H**	**1.21475700**	**2.02025700**	**0.06922800**

**C**	**-0.60603200**	**0.88994300**	**-0.10976700**
**H**	**-1.16600900**	**1.70424300**	**0.37765900**
**H**	**-0.70711600**	**1.05870100**	**-1.19599600**
**C**	**-1.24098200**	**-0.44340400**	**0.25293000**
**H**	**-0.68817800**	**-1.25712700**	**-0.24338600**
**H**	**-1.12097100**	**-0.61500300**	**1.33596700**
**C**	**2.51280000**	**-0.89895500**	**0.11289100**
**H**	**2.56012700**	**-1.01058200**	**1.20169000**
**H**	**3.12767100**	**-1.57623200**	**-0.48593500**
**C**	**1.74089800**	**0.02699600**	**-0.45222900**
**H**	**1.71834300**	**0.10115800**	**-1.54829500**
**C**	**-2.71147800**	**-0.51644100**	**-0.12114600**
**H**	**-3.15193300**	**-1.48621600**	**0.14895900**
**H**	**-3.28862200**	**0.26875900**	**0.38994100**
**H**	**-2.84919800**	**-0.37514400**	**-1.20369300**

Au3-(8)

0 2

C	-0.04672897	0.20560748	0.00000000
C	0.69431903	1.09923048	-0.42538800
H	-0.45872997	-0.72332152	0.36654600
Au	-3.93362897	2.71860548	0.03422100
Au	-1.96200897	4.65427048	-0.42309700
Au	-1.41708397	1.90331748	-0.18606900
C	1.80502503	1.91585848	-0.93369100
H	2.72616103	1.48276448	-0.51337300
H	1.85394803	1.76703748	-2.02200700
C	1.73187903	3.40614248	-0.61239400
H	2.61892803	3.88257648	-1.05655900
H	0.85289503	3.84307748	-1.11641800
C	1.67041703	3.72215648	0.87244900
H	0.76883103	3.25580348	1.30713300
H	2.53249903	3.25969548	1.38087600
C	1.64482403	5.21707048	1.14093100
H	0.76505203	5.68015448	0.66475700
H	1.59832703	5.43403448	2.21688900
H	2.54185003	5.70756148	0.73487500

Au3_out-(8)

0 2

C	0.28971962	1.32710284	0.00000000
C	-0.20494638	2.45969984	0.00011500
H	0.47442462	0.26308384	-0.00015000
Au	4.63448262	3.11273284	-0.00038900
Au	2.93340562	5.35823684	0.00027100
Au	2.02340462	2.69481884	0.00016300
C	-1.06902338	3.64439984	0.00026100
H	-0.82429738	4.25721484	0.88108200
H	-0.82432638	4.25741184	-0.88043100
C	-2.55083138	3.27221184	0.00023900
H	-2.76764038	2.65060684	0.88311800
H	-2.76765538	2.65077384	-0.88275400
C	-3.44378238	4.50223184	0.00036400
H	-3.20557238	5.12054384	-0.87995100
H	-3.20556438	5.12037284	0.88079700
C	-4.92115538	4.14917884	0.00033700

H	-5.18391738	3.55496884	0.88789000
H	-5.54937138	5.05014584	0.00043100
H	-5.18392738	3.55514584	-0.88733300

(8)

0 1

C	3.20061100	-0.50916200	0.00010900
H	4.15051200	-1.01572600	-0.00027000
C	2.13633200	0.06828300	-0.00007900
C	0.83539200	0.73178000	0.00000800
H	0.76972300	1.38989000	-0.88047300
H	0.76978500	1.38975500	0.88059100
C	-0.33790100	-0.24650600	-0.00001400
H	-0.26252900	-0.90249400	0.88201800
H	-0.26251400	-0.90246700	-0.88206800
C	-1.68107900	0.46442500	0.00000000
H	-1.73932000	1.12525100	-0.88013800
H	-1.73930300	1.12525300	0.88013800
C	-2.85493900	-0.49956400	0.00000600
H	-2.83072400	-1.14926800	0.88743400
H	-3.81543200	0.03358200	-0.00002100
H	-2.83069600	-1.14931500	-0.88738600

Au_3-(9)

0 2

C	-4.11766700	-0.02006700	1.99783100
H	-4.94860600	0.55567500	2.43377900
H	-3.61315800	-0.56547000	2.80701700
C	-4.66389700	-1.00308400	0.99106500
H	-5.36060100	-1.69295100	1.49968800
H	-5.23091000	-0.47575200	0.19893900
O	-3.58721400	-1.69555500	0.42687500
C	-3.95407000	-2.70968400	-0.46998700
H	-4.36836300	-3.57441700	0.07793400
H	-4.72533300	-2.34353400	-1.17140500
C	-2.73894700	-3.10431300	-1.26123600
H	-3.01844500	-3.83634900	-2.03897600
H	-2.34039100	-2.20359700	-1.76196800
O	-1.76642400	-3.62982400	-0.39978100
C	-0.47968900	-3.62408200	-0.95340300
H	-0.39732400	-4.35238100	-1.77931000
H	-0.24490400	-2.61975000	-1.35311400
C	0.51020000	-3.92179300	0.14780700
H	0.24261600	-3.30733300	1.02668500
H	0.48741100	-4.98488200	0.44388400
O	1.78072600	-3.55822800	-0.32461200
C	2.77258200	-3.52545100	0.66492500
H	3.09915200	-4.54370500	0.93680400
H	2.38263700	-3.03138400	1.57411500
C	3.94761100	-2.74772500	0.14359900
H	4.77568500	-2.80778000	0.86840000
H	4.28718200	-3.16731200	-0.81811600
O	3.57718500	-1.39791800	-0.03850100
C	4.64775400	-0.58287800	-0.46291500
H	5.05125600	-0.97618600	-1.41095900
H	5.44628900	-0.60218500	0.29666000

C	4.17987800	0.83105900	-0.65614400
H	5.06186400	1.46748200	-0.84879200
H	3.68401800	1.19994100	0.26292200
O	3.29167300	0.87710800	-1.73806000
C	2.82551900	2.17313100	-2.01377800
H	2.35200300	2.60645800	-1.11232700
H	3.66645000	2.82393200	-2.31206800
C	1.82575600	2.10491700	-3.13307400
H	1.61111700	3.12302900	-3.50265400
H	2.25539700	1.52121100	-3.96692900
O	0.65338400	1.49226400	-2.66953700
C	-0.18983600	1.04871100	-3.69591200
H	0.34286600	0.31565800	-4.32778000
H	-0.50223800	1.89134500	-4.33905700
C	-1.39943300	0.38549900	-3.10144400
H	-1.90898900	-0.20287600	-3.88585200
H	-1.07516700	-0.30405900	-2.29858900
O	-2.27911900	1.35012600	-2.58499000
C	-3.47683300	0.78621800	-2.12421300
H	-3.27781400	0.09689200	-1.27931400
H	-3.95732700	0.20314500	-2.93114800
C	-4.42248100	1.87363000	-1.69670600
H	-4.45314000	2.65378100	-2.47023500
H	-5.43192600	1.45185700	-1.57838000
O	-3.97751000	2.42657900	-0.46843700
H	-4.39857300	3.29142600	-0.30351900
O	-3.15837600	0.84893900	1.44295100
H	-3.55162700	1.46587900	0.76135000
Au	0.91066300	2.05306300	1.23570300
Au	-1.10071300	0.06876500	0.75583400
Au	1.33164000	-0.61003400	0.24740700

(9)

0 1

C	-1.87445300	-2.95215000	0.65704800
H	-2.64462800	-3.19551500	1.40733700
H	-1.54547600	-1.91786000	0.83163700
C	-0.70551100	-3.88513200	0.83825000
H	-0.41151600	-3.90301200	1.90344000
H	-1.00032100	-4.91465600	0.55758600
O	0.36429900	-3.45730600	0.04066900
C	1.43961600	-4.35638000	0.04942500
H	1.77799700	-4.54045400	1.08496300
H	1.12907200	-5.32403700	-0.38391900
C	2.57748200	-3.80311300	-0.75890300
H	3.32791600	-4.60157900	-0.89844500
H	2.21882200	-3.49501000	-1.75721800
O	3.15585800	-2.71548300	-0.08695800
C	4.33878600	-2.27585500	-0.70031700
H	5.04740100	-3.11857800	-0.78812300
H	4.13348800	-1.89801100	-1.71783700
C	4.98099800	-1.19882300	0.12575000
H	4.97939800	-1.50337500	1.18742500
H	6.03255600	-1.08517200	-0.19266800
O	4.30586000	0.02209700	-0.03676900
C	4.85999800	1.03265800	0.76467400
H	5.96068500	1.01909400	0.67347200

H	4.60700400	0.86411900	1.82665900
C	4.38738600	2.38929200	0.32853100
H	5.00219900	3.14501300	0.84960300
H	4.55140000	2.51189500	-0.75714000
O	3.03309000	2.58553600	0.64153700
C	2.65138800	3.92048400	0.43127200
H	2.81272800	4.20386600	-0.62421700
H	3.27208100	4.58577900	1.05804100
C	1.21008300	4.13963900	0.79080600
H	1.03799500	5.22948000	0.84095900
H	0.99535300	3.71723100	1.78860000
O	0.36774600	3.57256400	-0.17804000
C	-0.95834800	4.01853400	-0.05549800
H	-1.37015700	3.74664800	0.93253000
H	-0.99006700	5.11889200	-0.14587600
C	-1.80750200	3.41871400	-1.13893300
H	-2.75061200	3.98882000	-1.21453200
H	-1.28181400	3.50239900	-2.10697700
O	-2.08437000	2.07633600	-0.84300600
C	-2.79077400	1.42963800	-1.86960900
H	-2.12866600	1.24846100	-2.73454300
H	-3.63013500	2.06264800	-2.20936900
C	-3.33139900	0.12348200	-1.36365500
H	-3.70376500	-0.47133500	-2.21723500
H	-2.53325900	-0.46195400	-0.87699800
O	-4.37494100	0.38054200	-0.46076200
C	-4.90564500	-0.78433800	0.11116000
H	-4.12536100	-1.32151100	0.67787300
H	-5.27612400	-1.46360400	-0.67782700
C	-6.04345100	-0.42436400	1.02336500
H	-6.76368700	0.20462500	0.47595500
H	-6.55184800	-1.35298700	1.32784900
O	-5.52773300	0.25900600	2.14589400
H	-6.26316800	0.55696800	2.71653600
O	-2.40554400	-2.97638000	-0.64706000
H	-2.76152900	-3.86629100	-0.84177000

Path reaction with Au3 as a catalyst

Int1

0 2

Au	-0.55148273	1.49527989	-0.00721798
Au	-0.55148273	2.79568189	2.56422702
Au	-0.55148273	4.09608389	-0.00721798

Int2

0 2

C	0.47661869	0.55755395	0.00000000
C	-0.62044631	0.51434695	-0.56724700
H	1.37560869	0.86397495	0.51480600
Au	1.50476069	-3.93060505	-0.38600000
Au	-1.22776731	-3.91023105	-1.00724700
Au	0.28967369	-1.57825205	-0.45644700
C	-1.91129731	0.79816795	-1.21130200
H	-2.38233531	1.61024195	-0.63620800
H	-1.69497331	1.20184995	-2.21060100

C	-2.86123331	-0.38993205	-1.32720800
H	-3.73167831	-0.06053105	-1.91161500
H	-2.36879531	-1.18734305	-1.90787200
C	-3.30211131	-0.93537205	0.02140300
H	-2.41882531	-1.07222505	0.66787000
H	-3.92884031	-0.18324505	0.52991900
C	-4.03859331	-2.25113005	-0.03176900
C	-4.65953831	-2.71625205	-1.19356100
C	-4.09004031	-3.05608905	1.11211500
C	-5.30223331	-3.95352505	-1.21539000
H	-4.63503531	-2.11686205	-2.10663800
C	-4.73053531	-4.29016505	1.09588600
H	-3.60205631	-2.71039505	2.02884700
C	-5.33677431	-4.74755705	-0.07343600
H	-5.77540331	-4.29961505	-2.13780000
H	-4.75048331	-4.90362405	2.00021800
H	-5.83550931	-5.71955405	-0.09214500

TS1

0 2

C	0.00899281	-0.12589928	0.00000000
C	1.00551381	0.33929372	0.62132500
H	-0.08456819	0.02107572	-1.08234700
Au	-2.94336719	-3.52698728	1.36105200
Au	-3.30019119	-1.36224028	3.05465500
Au	-1.47831319	-1.34978628	0.78665500
C	2.28179381	0.98510872	0.86794200
H	2.59605681	1.26702772	-0.15414600
H	2.12983381	1.92065872	1.42315500
C	3.35632981	0.12796272	1.53790500
H	4.33354681	0.59447972	1.34731000
H	3.18596681	0.15473472	2.62128100
C	3.37302981	-1.32310028	1.07199400
H	2.37826581	-1.76201128	1.25210200
H	3.54841881	-1.37184628	-0.01370200
C	4.41004781	-2.13664028	1.79721400
C	4.23107181	-2.45379928	3.14896300
C	5.57881681	-2.56356628	1.16396500
C	5.18910381	-3.18354528	3.84555000
H	3.32130981	-2.12211528	3.66180300
C	6.54220781	-3.29353628	1.85773900
H	5.73472781	-2.32246028	0.10813800
C	6.35015781	-3.60618028	3.20035400
H	5.02824781	-3.42520628	4.89920400
H	7.44932581	-3.62095228	1.34339800
H	7.10378181	-4.18018028	3.74497000
O	0.77758181	-0.08890128	2.76443900
H	0.54575381	-1.04316528	2.90637400
H	-0.05045119	0.43794772	2.95607600
O	0.07970481	-2.72804728	3.21340700
O	-1.42574419	1.35053272	3.23163400
H	-0.89389819	-2.73039428	3.08165900
H	-2.10963819	0.68050472	3.47414700
C	0.64652981	-3.77510428	2.44881600
H	0.24537581	-4.75272428	2.75416400
H	0.46446581	-3.63047328	1.37088800
H	1.72818981	-3.76404728	2.63268800

C	-1.91131819	2.13037172	2.15481500
H	-2.33266819	1.49491272	1.35953800
H	-2.67432019	2.84367772	2.49814700
H	-1.05987819	2.68675272	1.74350800

Int3

0 2

C	-0.81834529	1.15107912	0.00000000
C	0.01650371	1.53305012	0.96405200
H	-0.49545329	1.45982212	-1.00141200
Au	-4.96221929	-1.14785188	0.18582500
Au	-3.74824129	-1.19795388	2.64299200
Au	-2.53684429	0.10870112	0.24141700
C	1.31488871	2.26891812	0.86532600
H	1.42510571	2.65071812	-0.15927300
H	1.28178171	3.13837412	1.53980600
C	2.51707471	1.39515812	1.23067700
H	3.40449771	2.03721912	1.32844500
H	2.35154671	0.94882912	2.22580300
C	2.77066871	0.30091812	0.20494100
H	1.81728271	-0.19141288	-0.04749500
H	3.11129271	0.76593512	-0.73607000
C	3.76122971	-0.76282288	0.60950300
C	4.71383071	-0.55834888	1.61128000
C	3.74423671	-2.00315588	-0.04043400
C	5.62573871	-1.55817388	1.94735100
H	4.75159571	0.39539512	2.14324400
C	4.65181371	-3.00316488	0.29113600
H	2.99851171	-2.18473688	-0.82121900
C	5.59974771	-2.78330088	1.28918400
H	6.36166871	-1.37492988	2.73447300
H	4.61646571	-3.96333688	-0.22993500
H	6.31275671	-3.56760388	1.55472800
O	-0.25828229	1.19171212	2.32266100
H	-0.27504329	0.11445912	2.45819000
H	-1.22583529	1.55439112	2.64395300
O	-0.39764429	-1.22916288	2.64506700
O	-2.46995329	1.90143712	3.10442600
H	-1.36991129	-1.41288788	2.72697400
H	-3.00926529	1.07235212	3.13873600
C	0.20557071	-2.10567088	1.69742800
H	0.08238971	-3.14460388	2.02593400
H	-0.23620429	-1.96236588	0.70065100
H	1.27440171	-1.86186588	1.66897600
C	-3.18987229	2.94154112	2.44847800
H	-3.55242129	2.60231512	1.46765200
H	-4.02851529	3.26248812	3.07777000
H	-2.49376729	3.77633012	2.31373700

Int4

-1 2

C	-2.09532365	0.23381295	0.00000000
H	-2.42860065	-0.77567805	0.27903500
C	-3.12525065	1.03402195	-0.29837700
O	-4.40504065	0.49620295	-0.22258400
H	-5.06896665	1.17810495	-0.43602600

Au	-0.09519265	0.52729595	-0.00115300
Au	2.62086935	0.38374095	0.42625500
C	-3.16410265	2.46726395	-0.76764500
H	-3.50950765	2.45776595	-1.81602100
H	-3.96466465	2.97978495	-0.20278500
C	-1.89662765	3.29623595	-0.66084600
H	-1.05611865	2.76091095	-1.12993700
H	-2.03713265	4.21764795	-1.24544100
C	-1.54224265	3.65712095	0.78128100
H	-1.63917665	2.76208795	1.41238800
H	-2.27957665	4.38856395	1.15313800
C	-0.15067365	4.21097495	0.93555400
C	0.76515635	3.61197095	1.80388300
C	0.27335335	5.31705195	0.18941700
C	2.06849935	4.09129995	1.91930300
H	0.45400435	2.73868795	2.38621300
C	1.57402235	5.80000795	0.29919700
H	-0.42671065	5.80803795	-0.49356600
C	2.47968935	5.18508795	1.16300000
H	2.76942535	3.59810695	2.59826700
H	1.88452235	6.66323095	-0.29532000
H	3.50242835	5.56091195	1.24693900
Au	1.92519535	2.18992895	-1.53573700

TS2

-1 2

C	-0.04496403	0.46762589	0.00000000
H	-0.83086703	0.08566289	-0.66952200
C	-0.03452103	1.86079689	0.06711400
O	-1.03243303	2.59833389	-0.29712900
H	-2.16617303	2.00105389	-0.06712000
O	-3.05798303	1.44879889	0.34109700
H	-2.51037303	0.73097589	1.21634600
O	-1.78590903	0.15533589	1.92000600
H	-0.84983703	0.28156489	1.21510600
C	-2.09626303	-1.21739811	2.05709300
H	-3.10051303	-1.32503311	2.48716900
H	-2.06442403	-1.73123011	1.08259500
H	-1.36760703	-1.68662211	2.73088400
C	-3.76846303	0.75300289	-0.65947800
H	-4.22702203	1.46575889	-1.35861700
H	-3.11301003	0.07278589	-1.22794600
H	-4.56805703	0.16213789	-0.19262600
Au	3.80412197	-2.27831111	0.62776000
Au	1.63718497	-0.65067511	0.28183400
Au	4.46387097	0.39749189	0.36379800
C	1.14930497	2.58425989	0.67476400
H	1.41540197	2.05879489	1.60871500
H	2.01698997	2.41233089	0.01708100
C	0.97228397	4.06884989	0.92912600
H	0.83582697	4.59445689	-0.02818000
H	0.04604997	4.24461289	1.49525700
C	2.13967597	4.67052689	1.70860100
H	2.14415397	4.25619089	2.72845400
H	1.96916997	5.75350289	1.82336200
C	3.50777297	4.45553289	1.10557600
C	4.59702797	4.13379589	1.92011000

C	3.73248697	4.58746689	-0.26932500
C	5.87269497	3.96093689	1.38683800
H	4.44085097	4.01635389	2.99687900
C	5.00314797	4.40916389	-0.80942700
H	2.89835697	4.83219989	-0.93352000
C	6.08085697	4.09757189	0.01754300
H	6.70722497	3.71006989	2.04668900
H	5.15313197	4.51538489	-1.88692500
H	7.07821497	3.95722189	-0.40621600

Int5

-1 2

C	-2.36429733	-0.17985611	-0.06191131
H	-2.52161933	-0.13991611	-1.14466731
C	-1.08862533	0.33410889	0.36296369
O	-0.88905733	0.78498089	1.51221569
H	-1.69485033	0.12797589	2.68529769
O	-2.09415233	-0.36857311	3.47123669
H	-1.27595333	-1.70742611	3.98438169
O	-0.88030833	-2.56982511	4.29431769
H	-3.20398133	0.23397589	0.50835269
C	-1.92723933	-3.49768811	4.40971269
H	-2.62908833	-3.43339111	3.55891069
H	-1.50109333	-4.51202911	4.40613169
H	-2.49756533	-3.36571911	5.34637269
C	-3.49919633	-0.35170311	3.42019069
H	-3.88361433	0.66700289	3.25173169
H	-3.88955233	-1.01347511	2.62701669
H	-3.88458533	-0.70490411	4.38657069
Au	-2.33650133	-2.24682511	0.42895069
Au	-3.35016833	-4.74444211	0.78927769
C	0.08457667	0.30673589	-0.59022731
H	0.73840767	1.14646089	-0.31560231
H	-0.26342733	0.46297489	-1.62030231
C	0.89006367	-0.99537311	-0.53064631
H	0.27678267	-1.82405311	-0.92303831
H	1.74797867	-0.88922711	-1.21204931
C	1.37503767	-1.34531211	0.86430169
H	0.50360767	-1.46591111	1.53195669
H	1.94864667	-0.49943511	1.27898269
C	2.20205767	-2.60229911	0.97253569
C	2.59575567	-3.04239611	2.24260069
C	2.56351967	-3.37576411	-0.13213231
C	3.31047867	-4.22239711	2.40735769
H	2.31756567	-2.44894211	3.11904869
C	3.28315767	-4.56087011	0.02730669
H	2.27105867	-3.06677111	-1.13805931
C	3.65480567	-4.99218311	1.29558369
H	3.59900367	-4.54786011	3.41012469
H	3.54928767	-5.15247611	-0.85245531
H	4.21393467	-5.92264011	1.42073869
Au	-0.60796833	-4.51869911	1.11751669

Int6

0 2

C	0.06294964	0.70143884	0.00000000

H	0.00629064	0.76994584	-1.08661300
C	1.24923564	1.10454584	0.60109900
O	1.35297664	1.34879284	1.88360400
H	0.59127764	0.90200884	2.44869200
O	-0.25475436	0.06664284	3.27300300
H	0.20032764	-0.82204816	3.43841100
O	0.91064364	-2.21541916	3.61653700
H	-0.87074136	0.83000084	0.55151600
C	0.26411464	-3.26316416	4.31683800
H	-0.58176336	-3.66601016	3.73731200
H	0.97172164	-4.07240016	4.54432400
H	-0.11176036	-2.84173016	5.25637800
C	-1.64001136	-0.12901816	3.07095900
H	-2.08739036	0.83776284	2.80775100
H	-1.82763836	-0.84519116	2.25332300
H	-2.11566136	-0.49509716	3.99174300
Au	0.49202464	-1.43028616	0.33383900
Au	-0.30159436	-3.95148916	0.72918100
C	2.50951664	1.36448684	-0.16863400
H	2.94765164	2.27929284	0.25718100
H	2.25764264	1.56577584	-1.21713900
C	3.54343164	0.23516884	-0.11319600
H	3.13906064	-0.64409016	-0.64328500
H	4.42256564	0.56834684	-0.68471600
C	3.95869164	-0.17172416	1.28930500
H	3.06549464	-0.45638916	1.86902200
H	4.38368564	0.69898584	1.81644100
C	4.93075264	-1.32328616	1.36659400
C	5.20518964	-1.89028916	2.61779400
C	5.54626664	-1.87923916	0.24336900
C	6.05522264	-2.98219216	2.74348600
H	4.72528264	-1.47017316	3.50756600
C	6.40127164	-2.97572916	0.36419400
H	5.35310064	-1.46785716	-0.74975500
C	6.65609464	-3.53418016	1.61162200
H	6.24821164	-3.41022516	3.73048300
H	6.86707564	-3.39706016	-0.53027600
H	7.32195064	-4.39539816	1.70544400
Au	2.45729264	-3.49736616	0.79662100
H	1.24453364	-2.55554516	2.75607500

TS3

0 2

C	-1.23201434	1.27697840	0.00000000
O	-1.69816334	1.01121240	1.12502900
H	-2.87332034	-0.05291260	1.23855200
O	-3.71815334	-0.57171660	1.03266500
Au	3.11835366	-1.90743260	0.30993700
Au	0.96825466	-0.66346560	-0.46917000
C	-0.74967634	2.67883740	-0.27776700
H	-1.20771034	3.33912140	0.47090000
H	-1.10516034	2.98298140	-1.27302400
C	0.77316166	2.81584840	-0.22912800
H	1.23499866	2.15962640	-0.98729600
H	1.02451466	3.84468640	-0.52306800
C	1.35514766	2.50246340	1.14484000
H	0.95745766	1.54097940	1.50922600

H	0.99900566	3.26140540	1.86254700
C	2.86108866	2.43013340	1.18298800
C	3.66132466	3.32603840	0.46613000
C	3.49562166	1.44075440	1.93976900
C	5.05052966	3.22866440	0.49700800
H	3.19659766	4.11337840	-0.13340100
C	4.88371466	1.33731240	1.97346000
H	2.88567666	0.72292640	2.49720100
C	5.66796366	2.22992040	1.24673400
H	5.65532166	3.93750740	-0.07433300
H	5.35454066	0.54757040	2.56499700
H	6.75757066	2.14938540	1.26607400
Au	3.53375566	0.23388840	-1.54094000
O	-3.82562134	0.17429940	-1.32521500
H	-3.92787334	-0.20538160	-0.34818000
C	-3.61987034	-1.92239560	1.44154600
H	-2.74795634	-2.41945660	0.98987800
H	-3.54837434	-1.98732760	2.53551700
H	-4.52994934	-2.43933260	1.11584700
C	-4.54661734	1.38599940	-1.49976800
H	-4.41409734	1.71521240	-2.53626700
H	-5.61187034	1.20477440	-1.31179400
H	-4.18126334	2.16688540	-0.81639500
C	-1.22747534	0.31924540	-1.07789100
H	-0.76129734	0.58789440	-2.03004100
H	-1.32169934	-0.75223260	-0.82862300
H	-2.57342334	0.31812840	-1.34167700
